# EvoMining reveals the origin and fate of natural product biosynthetic enzymes

**DOI:** 10.1099/mgen.0.000260

**Published:** 2019-04-04

**Authors:** Nelly Sélem-Mojica, César Aguilar, Karina Gutiérrez-García, Christian E. Martínez-Guerrero, Fancisco Barona-Gómez

**Affiliations:** Evolution of Metabolic Diversity Laboratory, Langebio, Cinvestav-IPN, Irapuato, México; ^†^​ Present address: Nuclear-Mitochondrial Interaction and Paleogenomics Laboratory, Langebio, Cinvestav-IPN, Irapuato, México.

**Keywords:** specialized metabolism, genome mining of natural products, EvoMining, scytonemin biosynthesis, evolutionary genomics

## Abstract

Natural products (NPs), or specialized metabolites, are important for medicine and agriculture alike, and for the fitness of the organisms that produce them. NP genome-mining aims at extracting biosynthetic information from the genomes of microbes presumed to produce these compounds. Typically, canonical enzyme sequences from known biosynthetic systems are identified after sequence similarity searches. Despite this being an efficient process, the likelihood of identifying truly novel systems by this approach is low. To overcome this limitation, we previously introduced EvoMining, a genome-mining approach that incorporates evolutionary principles. Here, we release and use our latest EvoMining version, which includes novel visualization features and customizable databases, to analyse 42 central metabolic enzyme families (EFs) conserved throughout Actinobacteria , Cyanobacteria , *Pseudomonas* and Archaea. We found that expansion-and-recruitment profiles of these 42 families are lineage specific, opening the metabolic space related to ‘shell’ enzymes. These enzymes, which have been overlooked, are EFs with orthologues present in most of the genomes of a taxonomic group, but not in all. As a case study of canonical shell enzymes, we characterized the expansion and recruitment of glutamate dehydrogenase and acetolactate synthase into scytonemin biosynthesis, and into other central metabolic pathways driving Archaea and Bacteria adaptive evolution. By defining the origin and fate of enzymes, EvoMining complements traditional genome-mining approaches as an unbiased strategy and opens the door to gaining insights into the evolution of NP biosynthesis. We anticipate that EvoMining will be broadly used for evolutionary studies, and for generating predictions of unprecedented chemical scaffolds and new antibiotics. This article contains data hosted by Microreact.

## Data Summary

1. Databases have been deposited at Zenodo; DOI: 10.5281/zenodo.1219709 (https://zenodo.org/record/1219709#.XBpzdMaVvCI).

2. Trees and metadata have been deposited in Microreact: GDH Actinobacteria , https://microreact.org/project/r1IhjVm6X?tt=cr;GDH Cyanobacteria , https://microreact.org/project/HyjYUN7pQ?tt=cr;GDH *Pseudomonas *, https://microreact.org/project/rJPC4EQa7?tt=cr;ALS Archaea, https://microreact.org/project/ByUcvNmaX?tt=cr;ALS Cyanobacteria , https://microreact.org/project/B11HkUtdm?tt=cr.

3. EvoMining code has been deposited in GitHub (https://github.com/nselem/evomining).

4. Docker container has been deposited in DockerHub (https://hub.docker.com/r/nselem/evomining/).

**Impact Statement**

EvoMining allows the study of expansion-and-recruitment events in enzyme families (EFs) in Archaea and Bacteria lineages, with the goal of providing both evolutionary insights and a genome-mining approach for the discovery of truly novel natural product (NP) biosynthetic gene clusters. Thus, by better understanding the origin and fate of gene copies within EFs, this work contributes towards the identification of lineage-dependent enzymes that we call ‘shell’ enzymes, which are ideal beacons to unveil ‘chemical dark matter’. We show that enzyme functionality is a continuum, including transition enzymes located between central and specialized metabolism. To exemplify these evolutionary dynamics, we focus on the genes directing the synthesis of the sunscreen peptide scytonemin, as the two key enzymes of this biosynthetic pathway can be considered as canonical examples of shell enzymes. We also show how evolutionary approaches are suitable for studying unexplored lineages, such as those belonging to the domain Archaea, which is systematically mined here for novel NPs. The release of EvoMining as a stand-alone tool will allow researchers to explore their own EFs of interest, within their own genomic lineages of expertise, by considering the lessons learned from this work.

## Introduction

Natural products (NPs), or specialized metabolites, are naturally occurring molecules widely used in medicine and in other applications [1]. NPs are typically encoded in biosynthetic gene clusters (BGCs) found in the genomes of a wide range of organisms. From sources as diverse as bacteria, fungi and plants, there are around 1800 NPs with their cognate BGCs experimentally characterized. This body of knowledge, contained in a community-driven hierarchical repository called the Minimum Information about a Biosynthetic Gene cluster (MIBiG) [2], allows the investigation of newly sequenced Archaea and Bacteria genomes as never before. Indeed, current availability of around half a million bacterial genomes in public databases (DBs) has not only invigorated research into NPs, but it has also prompted the development of novel genome-mining bioinformatic tools [3, 4]. The latter have evolved from simple sequence similarity searches of known biosynthetic enzymes, with an emphasis on the domains of polyketide synthases (PKSs) and non-ribosomal peptide synthetases (NRPSs) [5, 6], to genome-mining platforms that look into complete BGCs, such as the antibiotics and secondary metabolite analysis shell (antiSMASH) [7]. For instance, from only 6200 closed bacterial genomes, a total number of 32 584 BGCs have been identified using antiSMASH [8].

Known classes of NP BGCs are successfully predicted by antiSMASH; nevertheless, not all BGCs correspond with well-known biosynthetic enzyme classes. Within MIBiG there are 231 BGCs (12.7 %) classified as ‘other’, which indeed lack a PKS, NRPS or any of the other enzyme classes characteristic of specialized metabolism. Absence of known biosynthetic enzymes makes these BGCs ‘atypical’, hard to identify, relating them to the term ‘chemical dark matter’ [9]. An example of this scenario is provided by the BGC of the cyanobacterial sunscreen scytonemin [10], which includes ScyB and ScyA as the two key biosynthetic enzymes sustaining the synthesis of this specialized metabolite [11, 12]. Interestingly, ScyB and ScyA are distant homologues of glutamate dehydrogenase (GDH) and acetolactate synthase (ALS), respectively, which take part in the reversible oxidative deamination of glutamate to α-ketoglutarate and ammonia [13] and in the synthesis of branched-chain amino acids [14], respectively.

Therefore, despite the overwhelming amount of accurately predicted BGCs, there is still plenty of space to discover and prioritize novel biosynthetic systems. For example, in our previous work, it was estimated that EvoMining expanded antiSMASH predictions between 15 and 26 % in Actinobacteria genomes [15]. To address the problem of the limited novelty revealed by genome-mining approaches based on sequence similarity searches, we have previously introduced the use of evolutionary principles driving the emergence of NP BGCs [15, 16]. The latter gave place to EvoMining, which recapitulates enzyme evolutionary events as follows: any given enzyme family (EF) may undergo expansions [17] due to gene duplication, leading to paralogues, and/or horizontal gene transfer, leading to xenologues. The emerging extra gene copies may be retained in the genome when they provide an advantage, as they evolve into novel enzyme functions [18] serving as raw material for new metabolic pathways. Since genes involved in a metabolic pathway tend to cluster together in bacterial genomes, conserved genomic vicinity can be taken as an indication of related gene functionality [19], whereas phylogenetic reconstruction of these EFs can differentiate between conserved copies devoted to central metabolism [16], and expansions recruited into NP biosynthesis or other metabolic adaptations.

To date, EvoMining has been used to show the occurrence of conserved EFs that fulfill related biochemical functions with different physiological roles [16, 20]. It has also been used for the discovery of NP BGCs with unprecedented biosynthetic enzymes essential for the synthesis of arsenolipids in *Streptomyces* [15]. As a related but independent follow-up of EvoMining, we have very recently released a phylogenomic approach, termed CORe Analysis of Syntenic Orthologs to prioritize Natural product BGCs, or corason. This algorithm addresses the evolutionary relationships between BGCs, allowing one to comprehensively identify all genomic vicinities in which particular biosynthetic gene cassettes are found [21]. Based on similar evolutionary ideas to those embraced by EvoMining, the Antibiotic Resistance Target Seeker, or arts [22], exploits the fact that some antibiotics function by interfering with central metabolic enzymes and, therefore, antibiotic-producing bacteria have mechanisms of self-protection encoded in extra gene copies. Although examples of the discovery of novel biosynthetic systems using corason or arts remain to be reported, these approaches together with EvoMining add to the growing notion that evolutionary paradigms can aid in the discovery of antibiotics [23].

Although elegant in their simplicity, the aforementioned evolutionary ideas oversimplify a far more complex scenario in which different evolutionary histories can involve different metabolic origins and fates [24]. For instance, the boundaries of central metabolism are hard to define, as conserved or core enzymes of genomic lineages tend to differ broadly [25], even within closely related clades or organisms [17]. Additionally, not all extra copies of expanded enzymes are recruited into specialized metabolism, as previously highlighted as a criticism of EvoMining [3]. Some of the expanded EFs may remain in central metabolism providing a certain level of metabolic redundancy, as it is the case of pyruvate kinases in glycolysis [20] or ketol-acid reductoisomerases in the biosynthesis of branched-chain amino acids [26] in *Streptomyces* species. Alternatively, expanded EFs could serve other physiological roles related to morphological development [27] or may represent metabolic adaptations that involve the use of different cofactors as in GDH of Archaea species [13, 28]. Although EvoMining overcomes the latter caveats by prioritizing extra copies that are similar in sequence to enzymes from NP BGCs with genome neighbourhood or vicinity support, there is much to be understood about the evolution of enzymes during the assembly of BGCs directing the synthesis of NPs.

An open question along these lines is whether we can identify novel enzymes that define a completely new class of BGCs, rather than only identifying accessory or precursor-supply enzymes. To address this question, here we developed EvoMining to allow customization of its DBs. We then performed a systematic analysis of expansion-and-recruitment events in different Archaea and Bacteria lineages, including divergent taxa ( Actinobacteria , Cyanobacteria , *Pseudomonas* and Archaea). Selected results were visualized with corason [19], as a feature that can be integrated into EvoMining, depicting the evolutionary dynamics leading to new BGCs. We analysed, as a case study, the BGCs of scytonemin, a pigment exclusively produced by Cyanobacteria [10, 12]. Our results directed us to rethink EvoMining to incorporate ‘shell’ enzymes [29]. In contrast with core EFs, with a copy in every genome of a given lineage, shell EFs are defined by 50 % conservation. By better understanding the origin and fate of metabolic enzymes, we demonstrate that expansion-and-recruitment profiles of EFs are taxa dependent, and that these observations relate to shell enzymes, which have great potential for the discovery of novel NP BGCs.

## Methods

### EvoMining container

EvoMining version 2.0 was developed as a standalone comparative genome-mining tool using Perl as the coding language and Docker [30] as the packaging platform. Previous recommendations for the use of containers were adopted [31]. A simplified version of corason code, reported simultaneously [21], was included within EvoMining 2.0 container to allow visualization of the genomic vicinity. EvoMining dependencies including blast, muscle, Gblocks, FastTree, as well as Newick utilities, were wrapped in the EvoMining Docker container, available at the DockerHub as the container nselem/evomining [*Data 1*]. EvoMining 2.0 code and operational details can be consulted in the user manual available at GitHub (https://github.com/nselem/evomining/wiki) [*Data 2*] The increased performances obtained by these developments, including the biological insights that can be gained, are summarized in Table 1.

**Table 1.** EvoMining 2.0 novel developments and associated biological insights

	EvoMining 1.0	EvoMining 2.0	Main results
Tool	Consulting website	Stand-alone tool in Docker container	Users can visualize EFs using a colour code according to their metabolic origin and fate.
DBs	Fixed	Provided by the user	DBs are customizable.
Genome DB	Actinobacteria (230 genomes)	Actinobacteria (1244 genomes); Cyanobacteria (416 genomes); * Pseudomonas * (219 genomes); Archaea (876 genomes)	Expansion profiles are related to genome size. The total number of expansions is similar across lineages up to a genome size of 5 Mbp. After this threshold, * Pseudomonas * surpasses Actinobacteria , which in turn exceeds Cyanobacteria . There are no reported archaeal genomes in this size range (Fig. 2b).
Enzyme DB	Actinobacteria (106 families)	Comparative framework consisting of 42 conserved EFs	Construction of the Enzyme DB by identification of 42 conserved EFs in Actinobacteria , Cyanobacteria , * Pseudomonas * and Archaea (Fig. 2a). Expansions, recruitments and phylogenetic histories are lineage dependent (Fig. 3a). Families in the Enzyme DB are often part of the shell genome, i.e. they are shared by the majority, but not all, of the genomes in a lineage (Fig. 3b, c). Shell EFs show expansion-and-recruitment events. These two observations stand behind the current EvoMining paradigm, leading to the use of core metabolism and shell enzymes as key concepts.
NP DB	Manual curation before MIBiG existence (226 BGCs)	MIBiG (1813 BGCs)	BGCs from different taxa allowing tracking of the fate of metabolic enzymes within vertical lineages, but also after horizontal gene transfer (Fig. 1).

### EvoMining expansion-and-recruitment algorithm

Expansion-and-recruitment events of EFs are the first output of EvoMining. Expanded EFs consist of all enzyme copies obtained after a blastp search using as queries the seed enzymes (Enzyme DB) against a DB of genomes (Genome DB), with an *E* value of 0.001 and bitscore 100. For this work, we adopted our previous approximation to describe significant expansions [15, 16], defined as at least one genome with an enzyme copy number beyond the mean plus two standard deviations. The resulting expansions are presented by EvoMining in a heat plot (Fig. S1), available with the online version of this article), which pinpoints, for each EF, all the organisms that have extra copies and significant expansions. It should be noted that not all extra copies are expansions, but may nevertheless be EvoMining predictions (see Results and Discussion). An advice for users, evolutionary patterns are better appreciated after reordering organisms in the heat plot as they appear in a species tree. Expanded EFs are then blastp queried using an *E* value 0.001 against a DB of NP biosynthetic enzymes (NP DB) or MIBiG [2]. The enzymes obtained represent recruitments into specialized metabolism, highlighting as well enzyme recruitments whose metabolic fates cannot be predicted. The most conserved enzyme sequences within each expanded EF are identified by bidirectional best hits (BBH) against the Enzyme DB. These actions were systematized as part of the EvoMining algorithm.

In synthesis, the EvoMining expansion-and-recruitment algorithm works by identifying three classes of enzyme copies in expanded EFs: (i) highly conserved enzyme copies; (ii) known enzyme recruitments into NP biosynthesis; and (iii) extra enzyme copies that are not enzyme recruitments or conserved copies, with a metabolic fate to be defined. Phylogenies of EFs, as explained in the following section, are used to assign metabolic origin and fate to all enzyme copies retrieved by the expansion-and-recruitment EvoMining algorithm.

### EvoMining phylogenetic reconstruction-and-visualization algorithm

**Fig. 1.**
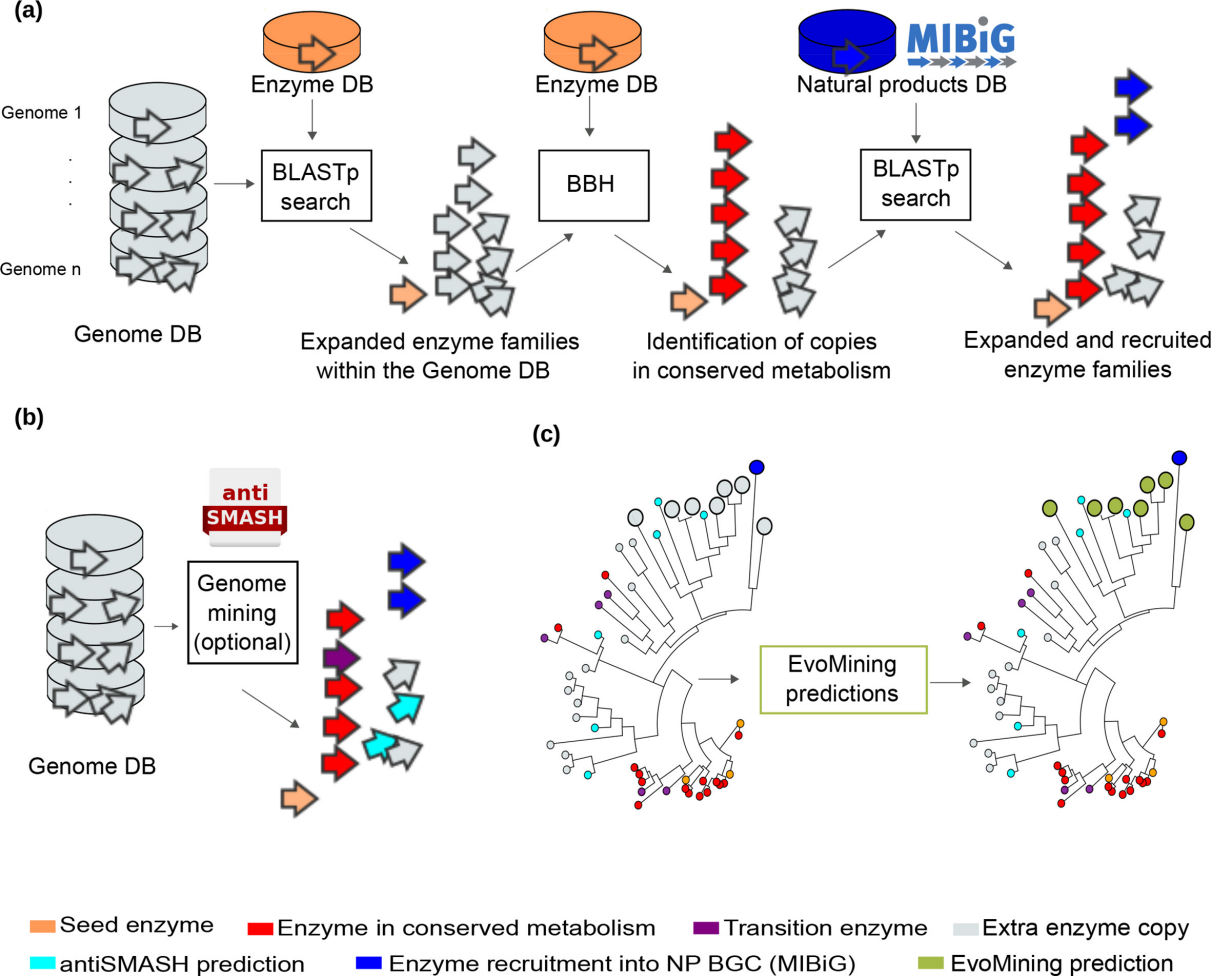
EvoMining pipeline, DBs and enzyme’s origin and fate. (a) EvoMining expansion and recruitment pipeline. Homologues and expansions of seed enzymes (orange) from the Enzyme DB are searched by blastp in the Genome DB. The outcome is integrated as the expanded EF. BBH of seed enzymes (red) are marked as conserved metabolism. The EFs are amplified after being compared against the NP DB (blue) to find recruitments defined as enzymes of the family that are part of an MIBiG BGC. (**b)** Optionally, antiSMASH predictions (cyan) can be added by the user. Enzyme predictions based on antiSMASH that are at the same time marked in red are defined as transition enzymes (purple). (c) EvoMining phylogenetic reconstruction and visualization algorithm. First, a phylogenetic reconstruction of an EF is carried out (left). Extra copies that are neither antiSMASH nor EvoMining predictions are left in grey. Second, on the right-hand side, EvoMining predictions (green), which are those extra copies closer to recruitments (blue) than to conserved metabolic enzymes (red), are shown. These predictions represent novel enzymes devoted to specialized metabolism.

EFs found to exhibit expansion-and-recruitment events were aligned with muscle v3.2 [32] and automatically curated with Gblocks v0.91b [33]. Parameters used included five positions as the minimum block length and ten as the maximum number of contiguous non-conserved positions. Positions with a gap in more than 50 % of the sequences were filtered and were not used for the final alignment. Curated alignments were phylogenetically reconstructed with FastTree 2.1 [34], which is an approximately maximum-likelihood method. These actions, leading to EvoMining trees per EF, were systematized as part of the EvoMining algorithm. Although EvoMining does not calculate by default antiSMASH predictions, these can also be provided by the user after running antiSMASH 3.0 [7] in every genome of the Genome DB as indicated in the user’s manual (Fig. 1(b)). Tree labelling with a colour code was automatized by the Newick utilities [35]. Enriched metadata, such as gene copy number per organism and functional information provided by the platform Rapid Annotation using Subsystem Technology (rast), in its classic version [36], was also added. Trees and metadata are arranged such that they are compatible with the specialized visualization tool Microreact [37].

EvoMining trees provide evolutionary insights into the metabolic origin and fate of members of any given EF by differentiating extra copies through a colour-labelling process (Fig. 1b, c). The most conserved sequences, identified after BBH using the Enzyme DB, are defined as conserved, or central metabolism copies, and these are marked in red. The well-known and experimentally supported recruitments into specialized metabolism, contained in MIBiG [2], are labelled in blue. Additionally, an EvoMining prediction is defined as enzymes that are closer to blue enzyme recruitments than to red conserved enzymes (Fig. 1c). These enzyme copies, coloured in green, represent the outputs with the largest potential to unveil unprecedented biosynthetic enzymes and their pathways. When antiSMASH results are provided, enzymes that belong to a BGC predicted by this algorithm, called antiSMASH predictions, are shown in cyan. When both EvoMining and antiSMASH call extra copies, cyan is used over green, restricting green to highlighting truly novel predictions. Purple is used for depicting transition enzymes, defined as sequences in the intersection between red (conserved metabolism) and cyan (specialized metabolism) labels. Finally, grey is used to highlight extra copies with an unknown metabolic fate.

### EvoMining DBs

Three DBs are needed to run EvoMining, the Genome DB, the Enzyme DB and the NP DB (or MIBiG). These DBs are provided as starting parameters through the command line before EvoMining is run. The integration of the current versions of these DBs is described as follows (Table 1).

#### Genome DB

The previous EvoMining Genome DB comprised 230 Actinobacteria genomes, including 50 different genera. In EvoMining 2.0, the Actinobacteria Genome DB was expanded to 1245 genomes, including 193 genera. Genome DBs for this work are available at zenodo with DOI: 10.5281/zenodo.1219709 [Data 3]. Additionally, three new Genome DBs were constructed and integrated, including data from organisms belonging to Cyanobacteria (416 genomes), *Pseudomonas* (219 genomes) and Archaea (876 genomes). These taxa were selected because BGCs from Actinobacteria (602 MIBiG BGCs), Cyanobacteria (60 MIBiG BGCs) and *Pseudomonas* (53 MIBiG BGCs) have been broadly characterized; but also to uncover novel metabolic space using genome mining approaches after looking into Archaea (1 BGC in new MIBiG version 1.4, none at time of first analyses using version 1.3).

Since EvoMining predictions are based on the ability of its algorithm to identify expanded enzymes, and not complete BGCs, draft genomes with a mean of five genes per contig were included. The selected genome sequences were retrieved from public DBs, as available in January of 2017 and functionally annotated by rast [36]. Genomes were mined by antiSMASH [7] with a parameter cf_threshold of 0.7 to identify enzymes belonging to a NP BGCs. These results were delivered as part of the final EvoMining trees through the internal DB called antiSMASH DB.

#### Enzyme DB

The previous EvoMining Enzyme DB comprised 106 EFs, which were selected on the basis of metabolic criteria, namely, enzymes from central metabolic pathways that could be unambiguously annotated within the genome-scale metabolic models of *Streptomyces coelicolor *, *Mycobacterium tuberculosis* and *Corynebacterium glutamicum* [15]. These 106 EFs comprised 339 actinobacterial amino acid sequences, used as seeds for our previous proof-of-concept analyses. In this version, the newly created Enzyme DB consists of a common set of conserved EFs identified in at least one seed genome from Cyanobacteria , *Pseudomonas* and Archaea. To avoid missing hits due to gaps in sequences, the seed genomes providing query enzymes were selected as they are contained in one single contig. For Cyanobacteria , these genomes were those from *Cyanothece* sp. ATCC 51142*, Synechococcus* sp. PCC 7002 and *Synechocystis* sp. PCC 6803; for the genus *Pseudomonas *, those from *Pseudomonas fluorescens* pf0-1, *Pseudomonas protegens* Pf5, *Pseudomonas syringae* and *Pseudomonas fulva* 12-X; and for the domain Archaea, those from *Natronomonas pharaonis *, *Methanosarcina acetivorans *, *Sulfolobus solfataricus* and '*Nanoarchaeum equitans*' Kin4-M. Seed enzymes are contained in the Enzyme DB, and their sequences were determined by selecting BBH using the previous Actinobacteria Enzyme DB [15] against the seed genomes of each lineage. BBH were found by using the Metaphor tool [38], discarding hits that did not account for at least 30 % of sequence identity across an alignment length of 80 % of the two protein sequences. The original 106 actinobacterial EFs were filtered to 42 EFs, which were shared by the seed genomes of Actinobacteria , Cyanobacteria , *Pseudomonas* and Archaea. Enzyme DBs for this work are available at zenodo with DOI: 10.5281/zenodo.1219709[Data 3]

#### NP DB

The original EvoMining NP DB included 226 manually curated BGCs [15]. In this work, the NP DB included in the analyses was MIBiG v1.3[2].The NP DB included in EvoMining container was updated to the release of August 2018, MIBiG v1.4, which includes 1813 NP BGCs and a total of 31 023 protein sequences.

### EvoMining analysis of EF from scytonemin BGC

After EvoMining analysis of the conserved 42 EFs, we focused on those present in the scytonemin BGC. Detailed analyses of this BGC included phylogenomic reconstruction and genomic vicinity visualization using corason [21]. Details of these EFs, including their distribution (Table S1, Fig. S2) and expansion trees (Figs S3–S9) are provided as supplementary material. Based on the results obtained by EvoMining, chemical diversity of the scytonemin BGCs was predicted utilizing their conserved enzyme repertoire, as revealed by corason, and the domain organization of the NRPS and NRPS-PKS assembly lines, using antiSMASH 3.0 [7] and PKS and NRPS analysis [6]. Up to 30 genes upstream and downstream the *scyA* gene were retrieved and analysed. For corason analysis, the amino acid sequences of ScyA and ScyB were concatenated and aligned using Muscle v3.2 [32]. A phylogenetic reconstruction was produced from the amino acid alignment matrix using MrBayes v3.2 [39], with a gamma distribution type range and 1 million generations. ScyA and ScyB sequences from '* Scytonema tolypothrichoides*' VB 61278 JXCA01 were used as the outgroup.

## Results and Discussion

### Increased applicability of EvoMining and validation strategy

To transform the website EvoMining version 1.0 into a genome-mining tool that allows analysis and visualization of large genomic datasets, we first aimed at customizing its DBs (Table 1). As a result, the three EvoMining inputs, (i) Genome DB, (ii) Enzyme DB (originally called PSCP, from Precursor Supply Central Pathways) and (iii) NP DB, can be modified, replaced or expanded by the user (Fig. 1a). For each EF, the pipeline produces an interactive, colour-coded tree of the expanded EF (Fig. 1b). Colours in the tree show information about the metabolic origin and/or fate of homologous enzymes present in the Genome DB. Red stands for central metabolism, which can be orange if it coincides with the seed enzyme; purple is used for transition enzymes; grey for expansions of unknown origin and/or fate; cyan for antiSMASH hits; blue for MIBiG recruitments; and green for EvoMining predictions. Visualization of the genomic vicinities in which each of these enzymes is encoded can be displayed. Thus, the newly incorporated changes allow, at a glance, exploration of the evolutionary dynamics of EFs, from central to different forms of specialized metabolism, with an emphasis on NP biosynthesis. Novel capabilities and potential insights provided by EvoMining are summarized in Table 1.

In order to validate EvoMining 2.0, in the following sections we first analyse whether expansion-and-recruitment rates of EFs are lineage dependent, and how this may relate to genome size. The concept of shell enzymes and its potential to unveil novel pathways is then presented. As further discussed, we define core EFs as those with a copy in every genome of a lineage, whereas shell EFs are defined as those enzymes that are shared by more than 50 % of the genomes of any given genomic lineage. Second, the scytonemin BGC is characterized as an example of the potential and intrinsic features of the EvoMining algorithm. The selection of this BGC follows the fact that it is composed of atypical biosynthetic enzymes that happened to be included within the 42 EFs analysed herein, namely, GDH and ALS. These EFs were detected by EvoMining as having originated in central metabolism, with their fate in specialized metabolism or central metabolic pathways related to adaptive physiologies involving different cofactors, depending on the taxon analysed.

### Enzyme expansion profiles are lineage dependent

**Fig. 2.**
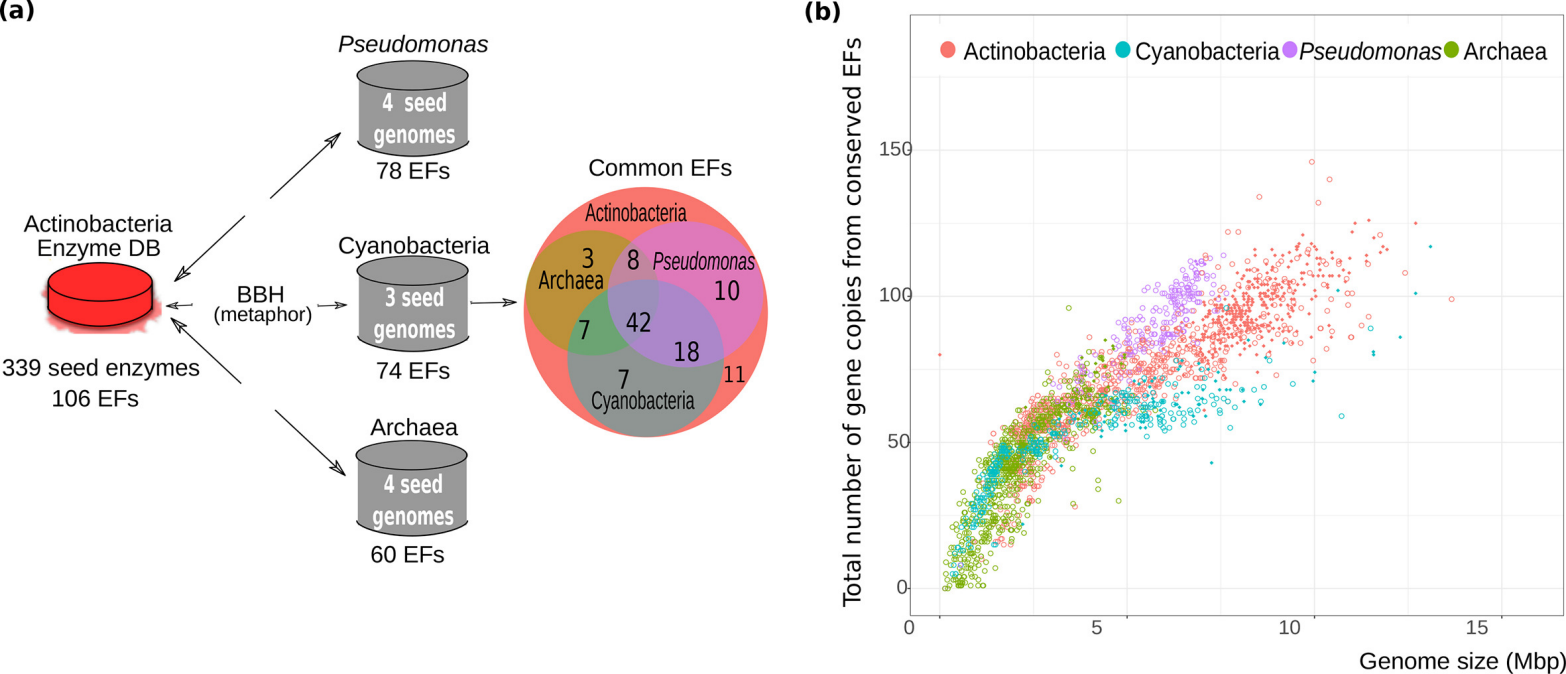
EvoMining Enzyme DB. (a) The previous EvoMining Enzyme DB was filtered to establish a common set of 42 conserved EFs for the phyla Actinobacteria and Cyanobacteria , the genus *Pseudomonas* and the domain Archaea. (b) All taxa show expansions of conserved EFs and these expansions correlate with genome size. Differences in expansion rates across taxa are principally noted after a genome size greater than 5 Mbp. At this threshold, *Pseudomonas* surpasses Actinobacteria expansions, which in turn exceeds Cyanobacteria .

To further gain insights into the evolution of enzymes and the pathways in which they take part, we exploited the taxonomic coverage of the selected lineages. The newly assembled Genome DB consisted of the phyla Actinobacteria and Cyanobacteria , the genus *Pseudomonas *, and the domain Archaea. Results shown in related figures are always presented following this order. The selection of these taxa is due to the possibility of analysing both well-known NP-producing micro-organisms, namely, Actinobacteria (602 MIBiG BGCs), Cyanobacteria (60 MIBiG BGCs) and *Pseudomonas* (53 MIBiG BGCs); but also poor NP-producing taxa, such as Archaea (0 BGCs in MIBiG version 1.3), which represents a domain whose NP biosynthetic capabilities have not been investigated until very recently [40]. Based on these Genome DBs, and following the scheme of Fig. 2(a), a set of EFs shared among them was first identified. Notably, only a fraction of the original 106 actinobacterial EFs was found conserved as new taxa were incorporated. Thus, each taxon-specific DB contains only 42 EFs (Table S1). The observation that 64 EFs are not conserved throughout these four taxa reflects on the species or lineage specificity of their metabolism [17], an intrinsic feature that is acknowledged and better exploited in this new version of EvoMining.

Using these conserved 42 EFs, we found that in all lineages the expansion profiles behave similarly until a genome size of 5 Mbp. After this threshold, the total number of sequences in the 42 EFs grows faster in the genus *Pseudomonas* than in the phylum Actinobacteria , which in turn surpasses the phylum Cyanobacteria and the domain Archaea (Fig. 2b). The latter observation may be related to the fact that there are no reports of Archaea genomes with sizes comparable to those typically found in *Streptomyces* or *Pseudomonas* (>5 Mbp). In contrast, Cyanobacteria , despite having large genomes, was the taxon with the fewest expansions. This could be a real biological observation, or that the selected Enzyme DB, by chance, lacks expansions in this lineage. In any case, these results suggest that EvoMining is well equipped to analyse large genomes when used as a tool to generate NP BGC predictions.

Our results also show that the taxonomic orders with the greatest number of copies in the common Enzyme DB were Streptomycetales and Nostocales , in Actinobacteria and Cyanobacteria , respectively. This observation coincides with the fact that these two orders have the largest genome size within their corresponding lineages, and these orders are well known to have metabolic diversity and biosynthetic potential. Interestingly, the class Halobacteria showed the largest number of expansions in Archaea, despite the fact that it is not the class with the largest genome size on average (Fig. S10). Yet, this result is in agreement with the observation that archaeocines, diketopiperazines, carotenoids and other NPs from Archaea were all isolated from Halobacteria species, even though their NP BGCs remained to be discovered [40]. Thus, EvoMining is a suitable tool to mine the genomes of unexplored lineages with the potential to encode truly novel pathways.

Overall, these results show that expansions from central families correlate with genome size. However, the increment of the expansion profiles is different in each genomic group, and this increment is not linear (Fig. 2b). These results are important to direct the use of EvoMining, first, as they emphasize the importance of a properly assembled and ad hoc Genome DB; and second, to better understand the predictions derived from its use when more than one genomic lineage, with different genome sizes, is explored. These points are revisited in the following sections.

### EvoMining reveals the occurrence of extra copies in shell enzymes

Having shown that divergent genomes experience different lineage-specific expansion profiles, we then focused on the expansion-and-recruitment patterns across the different taxa. Our analyses show that *Pseudomonas* has on average more copies per genome than any other taxa, as 54.8 % of the 42 EFs showed a maximum mean copy number for this lineage (Fig. S11). In contrast, Actinobacteria showed a maximum mean in only 26.2 % of the EFs, while Archaea and Cyanobacteria had a similar result, as little as 9.5 % (Table S1). While there are families like acetylornithine aminotransferase or ALS that are highly expanded in every lineage (coordinates A1 and E1, Fig. S11), many others exhibit differential behaviour. Such is the case of the fumarate reductase iron-sulfur subunit (coordinate C3, Fig. S11), which is highly expanded in Actinobacteria but has on average less than one copy per genome in Cyanobacteria .

**Fig. 3.**
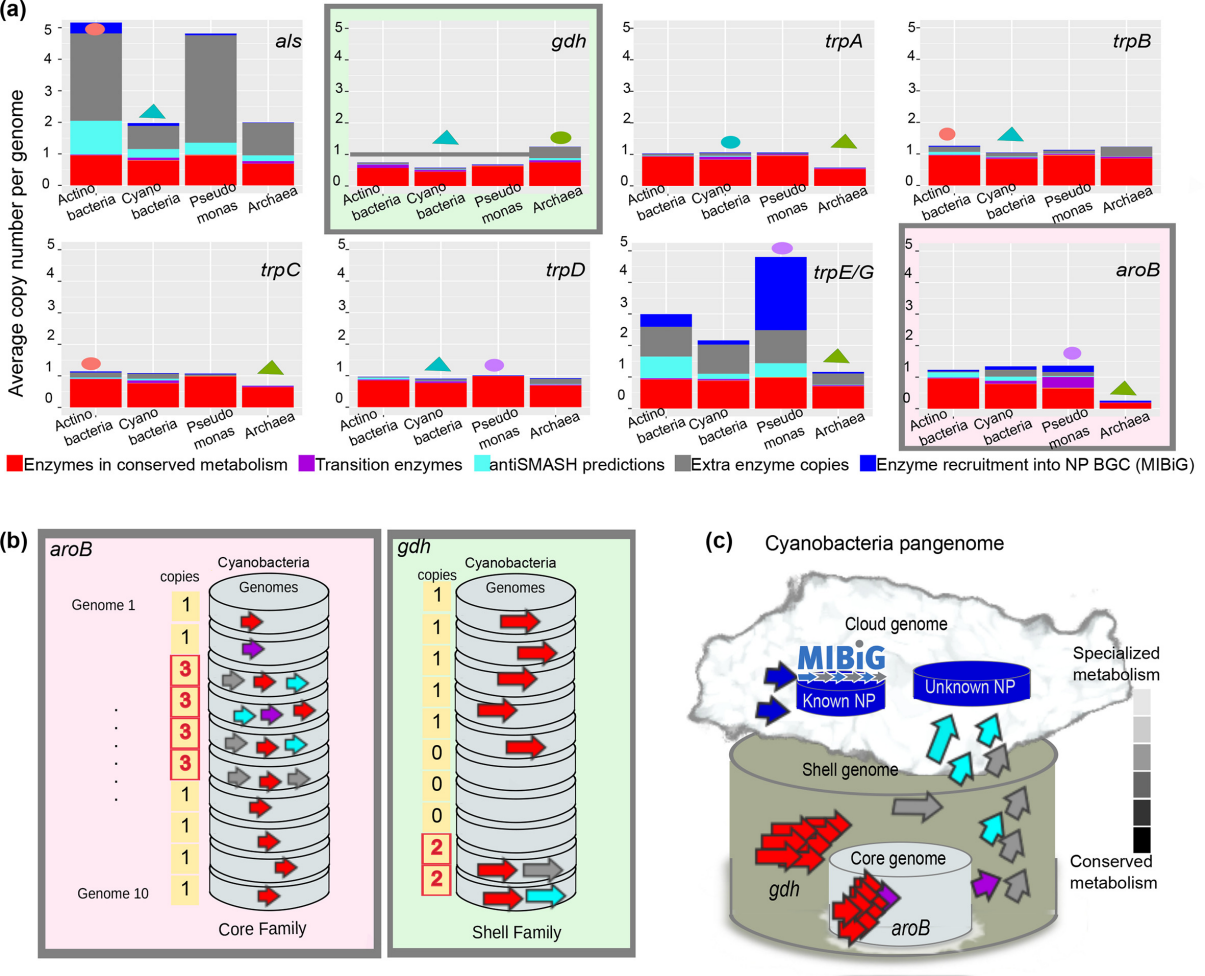
EvoMining profiles of selected conserved enzymes. (**a)** Expansion patterns of the eight conserved families whose extra copies participate in scytonemin biosynthesis. The full set of 42 EFs is shown in Fig. S11. Colour coding is as follows: red for conserved metabolism, blue for recruitments annotated at MIBiG, cyan for antiSMASH predictions of specialized metabolism, purple for the intersection between conserved metabolism and antiSMASH predictions, and grey for expansions without known metabolic fate. The order on the *x*-axis is Actinobacteria , Cyanobacteria , *Pseudomonas* and Archaea. Triangles indicate the lineage with the largest number of copies per genome on average, and circles stand for the least expanded lineage. Although Archaea tends to be the least expanded taxa, this tendency reverts in the GDH family. (**b)** An example of a core versus a shell EF is provided. AroB is a core EF because it has at least one copy per genome, while GDH is a shell EF because of its absence in three genomes. Despite being a shell EF, GDH has extra copies that can be recruited into specialized metabolism. (**c)** Model for the ‘cloud’ or variable genome composed partially by enzymes belonging to NP BGCs. In this model, conserved metabolism is composed of both shell and core EFs. These EFs may suffer from expansion events, and some of the extra copies are recruited to perform novel functions in specialized metabolism.

After inspecting these results in more detail, it was interesting to note that some EFs are not expanded. Indeed, even when the 42 EFs were found conserved throughout the seed genomes selected for each of the four taxa investigated, certain enzymes were not present in some of the genomes not used as seed organisms, but included in the Genome DB. Yet, the independent expansion profiles of each EF, in general, showed the same pattern as that recorded overall, with *Pseudomonas* as the lineage with the largest number of expansions, and Archaea with the least expansions. However, there are certain families that do not follow this trend, including some of the eight selected cases shown in Fig. 3(a). For instance, GDH is one of the four EFs with the largest number of expansions in Archaea. Moreover, GDH has less than one copy per genome in the other three taxa, to the point that this EF is not part of the core of these lineages. This contrasts with AroB, which shows extra copies and a mean copy number beyond one in all four genomic lineages analysed (Fig. 3b).

Based on these observations, we defined GDH as a member of the shell genome [29] of Actinobacteria , Cyanobacteria and *Pseudomonas *, consistent with the fact that it is not conserved but yet present in more than 50 % of the genomes of each of these lineages (Fig. 3a). Furthermore, as a consequence of this behaviour not unique to GDH, and to allow evolutionary analysis of selected enzymes within the scytonemin BGC, we focused on conserved EFs with extra copies beyond the mode. This criterion is different to that used for significant expansions, as defined in Methods, but allows calling for (shell) enzymes with interesting EvoMining profiles, opening the door to better understanding the origin and fate of biosynthetic enzymes. A conceptual scheme explaining the differential behaviours of GDH and AroB, where the differences between extra copies and significant expansions is emphasized, is provided in Fig. 3(b).

**Fig. 4.**
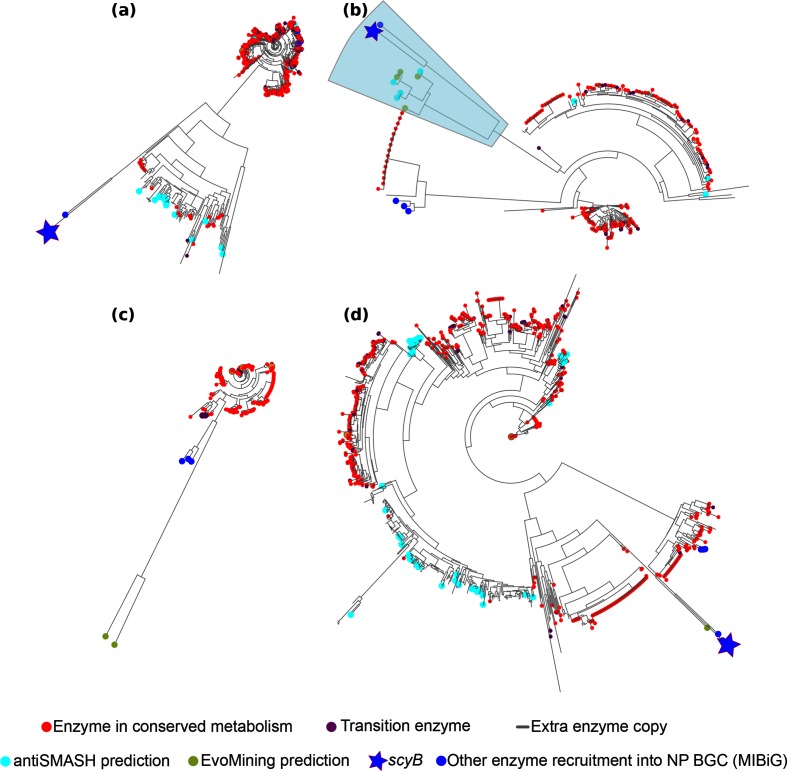
GDH EvoMining trees by taxa. (a) Lineage-specific phylogenetic reconstructions showing clear differences in expansion profiles in Actinobacteria , Cyanobacteria , *Pseudomonas* and Archaea. Actinobacteria has no EvoMining predictions, as its main expansion branch lacks MIBiG recruitments. Nevertheless, it is still possible that specialized metabolism may occur within those copies of unknown fate (grey). (b) Cyanobacteria possess four EvoMining predictions and four antiSMASH hits. *scyB* is located next to this specialized metabolism branch. (c) The majority of the *Pseudomonas* copies are labelled as conserved metabolism, with only two EvoMining predictions located in a divergent branch. *Pseudomonas* has a low mean copy number per genome, which is reflected in almost every copy labelled as central metabolism. (d) Archaea, the most expanded taxon, has a populated branch with expansions labelled as antiSMASH hits (cyan), but without any EvoMining prediction. The four lineages have MIBiG recruitments, but *scyB* is only shared between Actinobacteria , Cyanobacteria and Archaea. See also Fig. S12 for a combined visualization of these phylogenetic reconstructions that supports the occurrence of horizontal gene transfer as previously suggested [28].

We also found that our GDH EvoMining results included antiSMASH predictions for Actinobacteria , Cyanobacteria and Archaea, but not for *Pseudomonas *. This observation coincides with the fact that the recruitment of GDH by a NP BGC, namely, that of scytonemin, but also of the unrelated polyketide pactamycin [41], was only recorded for the former three taxa and not for *Pseudomonas* (see Fig. 4 in the following section). These results together suggest that the evolution of specialized metabolism is lineage dependent, but more importantly, that shell enzymes just as core EFs, possess the potential to drive the evolution of NP BGCs. Building up from these results (Fig. 3a, b), we provide a conceptual framework to explain how enzymes originate in conserved metabolism and evolve into specialized metabolism (cloud genome), via transition and/or shell enzymes in some cases (Fig. 3c). This model is relevant as the role of shell enzymes was previously overlooked when exploiting EvoMining as a genome-mining tool to generate novel biosynthetic predictions [21].

In the following section, we dissect the results provided by EvoMining for GDH by comparing phylogenetic trees obtained for each genomic lineage (Figs 4), as well as all together (Data 4 and Figs S12 and S13). In Archaea, GDH has on average 1.23 copies per genome, while in Actinobacteria , Cyanobacteria and *Pseudomonas* this mean is 0.74, 0.56 and 0.65, respectively. In the latter, GDH is part of the shell genome (Table S2). Thus, based on these observations, we specifically investigated the relationship between BGCs, expansion profiles and genomic lineages and, thus, we expanded our analyses to the ALS EF, which is one of the 42 conserved enzymes (Fig. 3a). Together with TrpA, TrpB, TrpC, TrpD, TrpEG and AroB (analysed in following sections), which are conserved amongst the 42 EFs, GDH and ALS have been recruited by the scytonemin BGC [11, 12], but not by the pactamycin BGC [41].

### EFs from the same BGC may experience different evolutionary dynamics: the case of GDH and ALS

The enzyme GDH, distributed in all domains of life because of both an ancestral origin and horizontal gene transfers [28, 42] (Fig. S12), catalyses the reversible oxidative deamination of glutamate into α-ketoglutarate and ammonia. This EF exists in three classes according to their use of cofactors. The first class uses NAD+ and it is referred to as GDH(NAD+). The second class utilizes NADP+ and it is known as GDH(NADP+). And the third class uses both NAD+ and NADP+; it is, therefore, referred to as GDH(NAD+ and NADP+) [13]. Moreover, GDH enzymes are very diverse and can be divided according to their taxonomic distribution and structural features [28]. However, although this GDH classification reflects more closely on the evolutionary history of this enzyme, for the sake of simplicity we adopted for our analysis the three GDH classes according to their cofactor specificity. GDH(NAD+) is utilized for glutamate oxidation and GDH(NADP+) for fixing ammonia, although some enzymes from Archaea can perform equally well with both cofactors [13]. NAD or NADP specificity has probably emerged repeatedly, as it has been shown that a few mutations can reverse specificity [42]. This suggests that global sequence similarity does not indicate similar specificity, which is an important consideration when analysing highly divergent EFs from different genomic lineages.

A detailed examination of the GDH EF showed that expansion events are not abundant in Actinobacteria [Data 5] (Fig. 4a), or in Cyanobacteria [Data 6] (Fig. 4b), and that they are mostly absent from *Pseudomonas [Data 7]* (Fig. 4c). In contrast, a significant number of expansions were found in Archaea [Data 8] (Fig. 4d). Thus, we focused on Archaea, and performed a detailed annotation to make sense of the resulting GDH EvoMining tree, which was rooted with a seed sequence from *Sulfolobus *, predicted to be a dual NAD(P)+ utilizing enzyme [43]. Notably, the three GDH classes alternate throughout the tree according to rast annotation (Fig. S13, Data 5-8). As expected, most of the sequences classified as central metabolic enzymes were situated in the early or basal branches of the tree. A more divergent and larger clade, consisting almost exclusively of NAD(P)-specific enzymes [44], included many expansions that are antiSMASH hits, with only two central metabolic enzymes. Functional annotation of the genomic vicinity of these GDH orthologues points towards a potential recruitment by specialized metabolism. These recruitments were identified mainly in organisms from the genera *Haladaptatus *, *Haloterrigena *, *Natrialba *, *Natrinema *, *Natrialbaceae* and *Natronococcus *. The coding genes are found within a genomic context suggestive of the synthesis of terpenes, as it includes enzymes related to geranyl pyrophosphate, a precursor to all terpenes and terpenoids [45]. Lastly, despite its higher divergence, the following two major branches in the tree also correspond to enzymes devoted to central metabolism (Fig. 4d).

In contrast with the broadly occurring GDH expansions related to metabolic adaptations in Archaea, the resulting Cyanobacteria tree showed expansions only in 4.5 % of the genomes (Fig. 4b, Table S2). Among these expansions, four antiSMASH hits and four EvoMining predictions confirmed the branch that contains ScyB enzymes, which are GDH homologues. ScyB participates in the synthesis of scytonemin, a yellow sunscreen pigment produced by many Cyanobacteria to protect them against UV-A radiation [11]. '* Nostoc punctiforme *' PCC 73102 is the scytonemin producer deposited at MIBiG. Unexpectedly, EvoMining only revealed a few GDH sequences from *Nostoc* species, even though s*cyB* homologues can be found in their genomes, as will be further discussed in the final phylogenomics section. This observation could be due to large sequence divergence between copies devoted to central and/or specialized metabolism in these organisms, despite them being closely related.

**Fig. 5.**
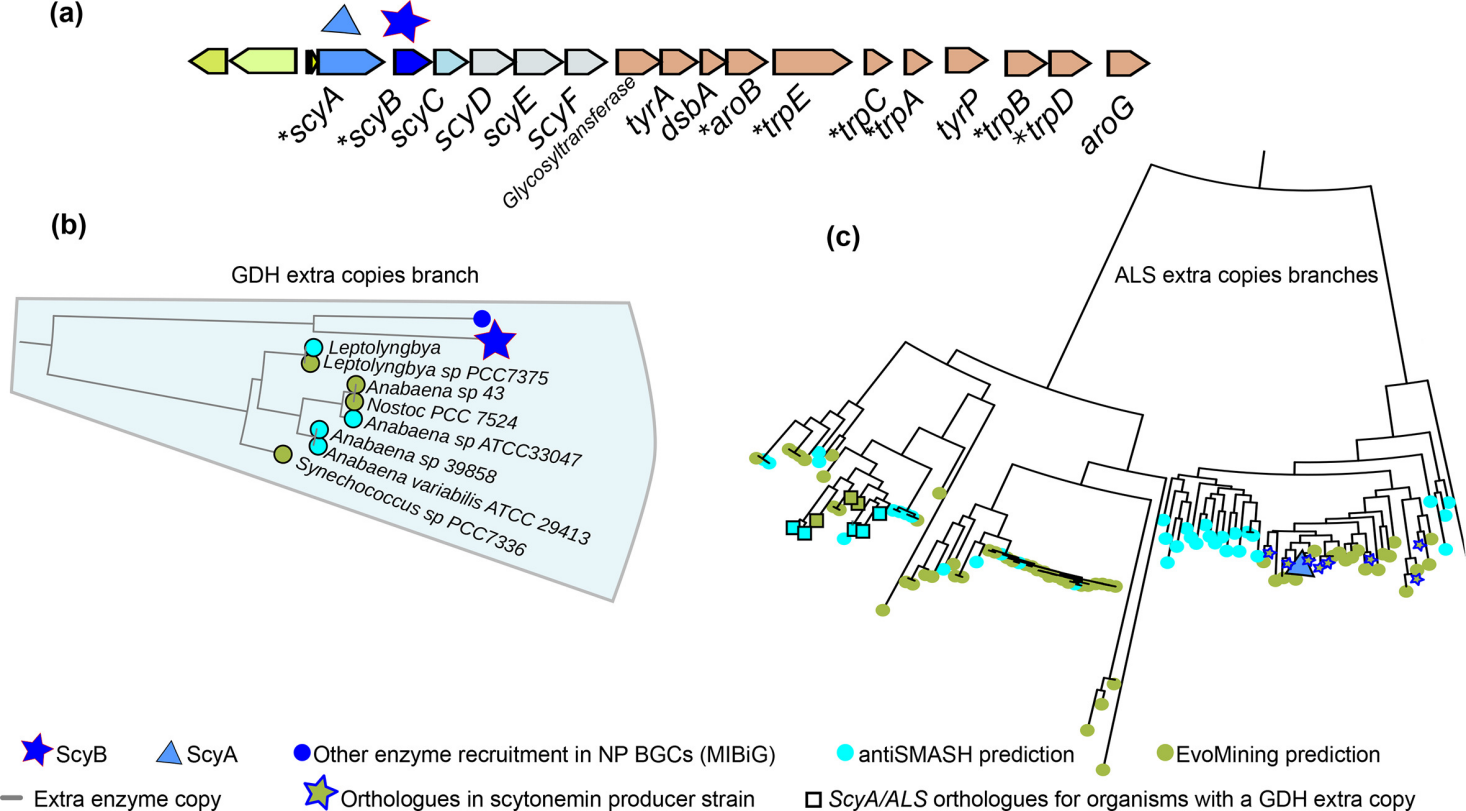
GDH and ALS recruitments by the scytonemin BGC. (a) Scytonemin BGC from '* Nostoc punctiforme *' is composed of regulatory genes (green), genes that participate in scytonemin biosynthesis (blue) and genes devoted to precursor supply (brown). Eight EFs of the scytonemin BGC were found to have their origin within the 42 conserved EFs, as shown by asterisks. (b) Detailed view (zoom-in) of Cyanobacteria GDH expansion branch close to ScyB. Unexpectedly, many of the known scytonemin producers are not found in this branch. (c) Zoom-in of the ScyA branch, showing ALS expansions correctly and exclusively marked by EvoMining with a fate in specialized metabolism. Known scytonemin producers are marked with stars. Squares indicate expansions devoted to specialized metabolism located in the genomic vicinity of GDH expansions that coincide with the ScyB branch. Cyanobacteria EvoMining trees of *trpA*, *trpB*, *trpC*, *trpD*, *trpE* and *aroB* are available in Microreact (Table S3).

Analysis of the organization of the scytonemin BGC made us realize that the *scyB* gene is always next to the *scyA* gene (Fig. 5a). This gene encodes a homologue of the ALS large subunit, an enzyme that was included amongst the 42 EFs analysed herein, with a mean copy number of 1.87 % in the entire Cyanobacteria DB, and a mean of 2.1 copies in organisms with at least a copy, but with a statistical mode of 1 (Table S2). This data is indicative that many organisms have more than two ALS copies, which may correlate with the fact that this family showed larger dispersion around the mode (Fig. S4, blue line). After analysis of the Cyanobacteria ALS EvoMining tree (Fig. S11 and Data 9), it was found that *scyA* is in fact a recruitment localized in the same branch that contains ALS sequences from *Nostoc* spp., which were labelled as EvoMining predictions (Fig. 5c). The latter predictions actually include more than 20 organisms that are known to be scytonemin producers [10, 12], an observation that agrees with the fact that the sister branches in the ALS tree show antiSMASH hits. Interestingly, organisms in this branch correspond to the same organisms revealed after the EvoMining analysis of GDH, which can be seen in the detailed zoomed-in view of the ScyB branch (Fig. 5b).

These results together suggest co-diversification, via expansion-and-recruitment events, of ScyA and ScyB from ALS and GDH, respectively. However, it should be noted that the expansion profiles of these EFs were quite different. Indeed, the sequence similarity between the GDH EF and homologues of ScyB turned out not to be enough to reconstruct a ScyB branch with enough expansions to suggest the occurrence of an NP BGC. This contrasted with the scenario found when ALS and ScyA were analysed. These results provide important lessons when using EvoMining as a genome-mining tool, as enzymes that co-evolve may be subject to different constraints and evolutionary rates.

### Phylogenomics analysis of the scytonemin BGC

**Fig. 6.**
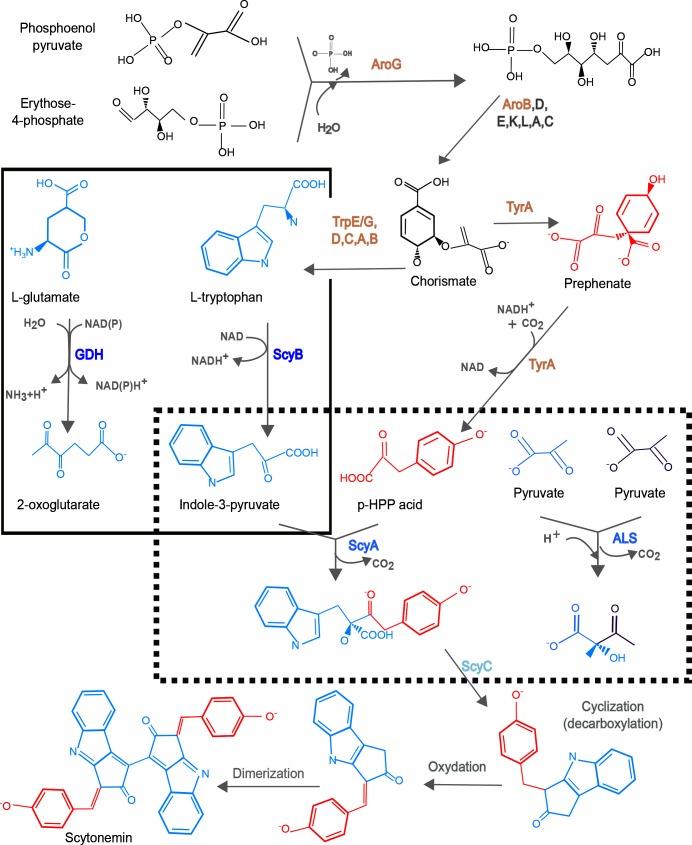
Metabolic origin and fate of GDH/ScyA and ALS/ScyB and scytonemin biosynthesis. AroG and AroB participate in the synthesis of chorismate, an intermediary that is transformed in the precursors leading to ScyA substrates, i.e. l-tryptophan and prephenate. The reaction catalysed by ScyB converting tryptophan into indole-3-pyruvate is similar to the conversion of l-glutamate into 2-oxoglutarate, catalysed by GDH (square with a solid outline). ScyA catalyses the decarboxylation of indole-3-pyruvate and *p*-hydroxy-phenyl-pyruvic acid (p-HPP) to form a dipeptide that serves as a scytonemin precursor. This reaction is analogous to the decarboxylation of two pyruvates by the original ALS enzyme (rectangle with a dotted outline). ScyC performs a cyclization followed by oxidation and dimerization steps that conclude with the scytonemin pathway. Enzymes in the scytonemin BGC devoted to synthesis of precursors are coloured in brown and scytonemin biosynthetic enzymes are coloured in blue.

The most characterized scytonemin BGC, shown in Figs 5(a) and 6, consists of 18 genes [12]. In addition to regulatory genes, this BGC includes the main biosynthetic genes, *scyABC*; tailoring-enzyme genes involved in late dimerization and oxidation steps, *scyDEF*; and in some cases, genes involved in precursor supply, namely, *tyrA*, *dsbA*, *aroB*, *trpE/G*, *trpC*, *trpA*, *tyrP, trpB*, *trpD*, *aroG*. The enzymes TrpABCDEG and AroB are part of the aromatic amino acid and shikimic acid pathways, and they seem to provide precursors for the synthesis of scytonemin in the form of l-tryptophan and prephenate. Interestingly, ScyA and ScyB have an origin in central metabolism, as they are homologues of ALS and GDH, but they have evolved different substrate specificities (Fig. 6). ALS joins two pyruvates, leading to S-2-acetolactate [14], while ScyB joins indole-3-pyruvate with *p*-hydroxy-phenyl-pyruvic acid. Analogously, but in central metabolism, GDH converts l-glutamate into 2-oxoglutarate [13], while ScyA catalyses an oxidative deamination of tryptophan. The product of these two enzymes, acting sequentially, is a dipeptide, which is cyclized by ScyC. The pathway finishes with a series of oxidations and a dimerization step to yield scytonemin [11].

In addition to GDH and ALS, 6 of the EFs that are present in the scytonemin BGC are part of the 42 EFs analysed herein (Fig. 3a, Table S3). All of them have an origin in central metabolism and have been recruited into the scytonemin BGC (Figs 5 and S3–S9). From these, six of the seven EvoMining trees contain expansions that turned out to be EvoMining predictions, as the expansion branches include scytonemin genes, an indication of specialized metabolism. These families include AroB, as well as all of the genes of the l-tryptophan biosynthetic pathway, other than *trpF.* The EvoMining predictions include enzymes from other sunscreen biosynthetic systems, such as shinorine and mycosporine-like amino acids [46] (Fig. S8), as well as other unrelated NPs, including welwitindolinone [47], ambiguine [48] and fischerindoline [49] (Figs S3–S7). These results illustrate how EvoMining can complement antiSMASH by identifying sequences that belong to non-traditional NP BGCs, even when substrate specificity has not been changed. To further investigate the presumed co-evolution of ScyA and ScyB, we reconstructed the evolutionary history of these enzymes by concatenating their sequences, and contrasting the resulting phylogenetic reconstruction with the genomic vicinity of their cognate BGC, using corason [21]. The resulting phylogenomic analysis revealed 34 cyanobacterial organisms with chemical diversity around their scytonemin BGC.

**Fig. 7.**
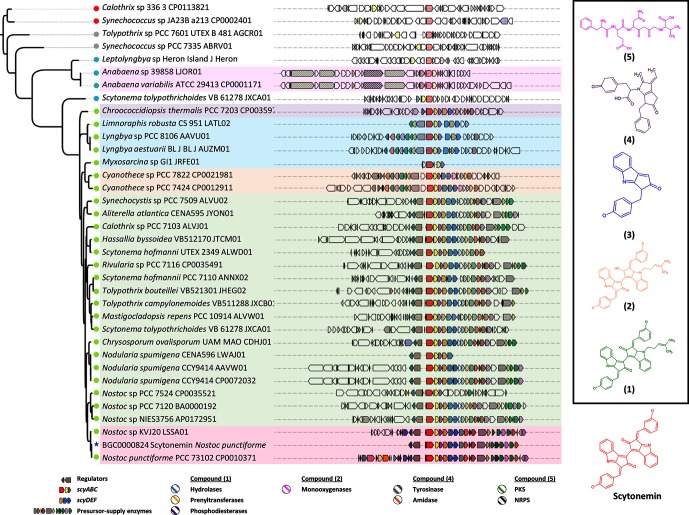
Phylogenomic analysis of *scyA* and *sycB* showed chemical diversity around the scytonemin BGC. Genomic vicinities containing both *scyA* and *scyB* in Cyanobacteria are shown next to a phylogenetic reconstruction using the protein sequences of these two genes. EvoMining classification for the ALS family is shown in a circle. Relevant accessory genes in variants of scytonemin BGC, highlighted by coloured boxes that define phylogenetic clades with biosynthetic signals, are labelled as follows: hydrolases (blue pattern), prenyltransferases (yellow pattern), phosphodiesterases (purple pattern), monooxygenases (pink pattern), tyrosinases (grey pattern) and amidases (orange pattern). The NRPS and PKS from the basal branches are coloured in green and black patterns, respectively. Five putative chemical structures related to scytonemin were predicted considering biosynthetic logics and gain-and-losses of key genes, as shown in the hand-right panel of the figure.

We could predict five additional putative chemical structures related to scytonemin, which correlate with episodes of gene loss-and-gain at these loci (Fig. 7). The incorporation of genes that encode other enzymes, such as hydrolases (blue pattern), prenyltransferases (yellow pattern), phosphodiesterases (purple pattern) and monooxygenases (pink pattern), could be forming the congeners of scytonemin compounds 1 and 2. Moreover, gene losses related to the enzymes ScyDEF and the appearance of other enzymes, such as tyrosinase (grey pattern) and/or an amidase (orange pattern) might be driving the synthesis of compounds 3 and 4. We also found that *scyA* and *scyB* are part of a BGC that contains a hybrid NRPS-PKS (green and black pattern, respectively). Following biosynthetic logics related to these enzymes, a predicted compound 5 is proposed. The chemical diversity suggested by these predictions, which can only be validated by further experimental characterization, emphasizes the evolutionary dynamics of specialized metabolism. Notably, these dynamics were traceable by means of using ScyA and ScyB as sequence beacons. These results suggest the increased predictive power of EvoMining for opening new metabolic spaces typically overlooked by standard NP genome mining approaches.

### Final remarks and considerations for the use of EvoMining

EvoMining was developed as a stand-alone genome-mining tool, and it was applied to a selected Enzyme DB composed of EFs common to highly divergent phyla. Our analyses lead to the conclusion that expansion-and-recruitment events are both EF and genomic lineage dependent, an important consideration when using EvoMining. Although genome size seems to matter, we also found exceptions where EvoMining could predict novel BGCs in relatively small genomes, suggesting that further analyses are needed to assess this relationship. Along these lines, we opted to compare genomic lineages that are not only highly divergent, and in some cases poorly understood with regards to NP biosynthesis, but also disproportionate in terms of their taxonomic resolution and distances. Thus, it is possible that these factors could have imposed a bias when establishing relationships between genome size, the rate of gene expansions and metabolic diversity.

After comprehensively analysing GDH, an EF notably expanded in Archaea but not in other taxa, we provide an example of a recruitment of a central metabolic enzyme by a NP BGC, as well as by other metabolic pathways. It is interesting to note that the most expanded EF in previous EvoMining proof-of-concept analyses [15] were asparagine synthase, 2-dehydro-3-deoxyphosphoheptanoate aldolase and 3-phosphoshikimate-1-carboxivinyl transferase, which lead to the discovery of unprecedented arsenolipid biosynthetic enzymes. Notably, none of these enzymes were part of the 42 EFs analysed herein, reinforcing the notion that not only conserved enzymes, but also shell enzymes with extra copies, can serve as beacons for the discovery of novel NP BGCs. These observations emphasize the predictive nature of EvoMining, which became apparent only after the origin and fate of enzymes could be traced back to evolutionary events at different levels, from genome dynamics involving large loci, to different mutations rates at the protein sequence level.

EvoMining users, therefore, should define beforehand the most appropriate EF to be used for a certain taxonomic group. The selected Enzyme DB should contain a set of EFs where expansion patterns could be detected. In turn, EFs with a distribution restricted to a small percentage of genomes are not suitable for EvoMining analysis. It is also important to determine which EFs are shared by most of the genomes within the genomic lineages of interest, and whether this is important for the type of EvoMining analyses to be performed. The original EvoMining DB included manually curated EFs only involving central metabolic enzymes, but, as it was demonstrated here, these did not necessarily represent the core enzymatic repertoire of Actinobacteria . This relates to the difficulty of defining what is central metabolism; thus, we prefer to use the term core enzymes at different thresholds of conservation. In our case, we used 50 % to define shell enzymes. This notion implies the possibility of automatizing Enzyme DB integration by selecting for EFs in any given genomic lineage, avoiding the need to arbitrarily define what is central metabolism.

Another key point in EvoMining success relates to the improvement of the NP DB due to the availability of MIBiG [2]. The previous EvoMining version did not include a cyanobacterial NP BGC, and for this reason only this EvoMining version could identify hits for the ScyA and ScyB branches. Nevertheless, in the absence of the signals provided by MIBiG, the extra copies of these EFs would have been marked by EvoMining as expansions not involved in central metabolism. This maybe the case in Archaea, where some sequences in the GDH tree are labelled as such, possibly related to terpenes as well as to other metabolic fates yet-to-be discovered. The presence of only one Archaea BGC at MIBiG is clearly due to the limited research available of the potential of Archaea to synthesize NPs, as our results suggest that current methods based on previous knowledge from unrelated taxa impose biases that hamper our ability to unlock the metabolic diversity of this domain of life. We anticipate that this situation will be overcome by EvoMining, as it is a less-biased and rule-independent approach.

## Data Bibliography

Nelly Sélem-Mojica. EvoMining Docker container. DockerHub: (https://hub.docker.com/r/nselem/evomining/).
Nelly Sélem-Mojica. Code of EvoMining pipeline. Github (https://github.com/nselem/evomining).
Nelly Sélem-Mojica. EvoMining databases are deposited at Zenodo with DOI: 10.5281/zenodo.1219709. (https://zenodo.org/record/1219709#.XBpzdMaVvCI).
4. Nelly Sélem-Mojica. Joined EvoMining GDH tree of Actinobacteria, Cyanobacteria, *Pseudomonas* and Archaea. Microreact: https://microreact.org/project/SJw7zVs1V?tt=cr
Nelly Sélem-Mojica EvoMining tree of GDH in Actinobacteria . Microreact: https://microreact.org/project/r1IhjVm6X?tt=cr
Nelly Sélem-Mojica. EvoMining tree of GDH in Cyanobacteria . Microreact: https://microreact.org/project/HyjYUN7pQ?tt=cr
Nelly Sélem-Mojica. EvoMining tree of GDH in *Pseudomonas .* Microreact: https://microreact.org/project/rJPC4EQa7?tt=cr
Nelly Sélem-Mojica. EvoMining tree of GDH in Archaea. Microreact: https://microreact.org/project/ByUcvNmaX?tt=cr
Nelly Sélem-Mojica. EvoMining tree of ALS in Cyanobacteria . Microreact: https://microreact.org/project/B11HkUtdm?tt=cr

## Supplementary Data

Supplementary File 1
