# Supplementary material for: EvoMining reveals the origin and fate of natural product biosynthetic enzymes
Source: Microb Genom. 2019 Apr 4;5(12):e000260. doi: 10.1099/mgen.0.000260 (PMC6939163; doi:10.1099/mgen.0.000260)
Supplement: Supplementary File 1 [file mgen-5-260-s001.pdf]

Supplementary Information

EvoMining reveals the origin and fate of natural products biosynthetic enzymes

Nelly Selem-Mojica, César Aguilar, Karina Gutiérrez-García, Christian E. Martínez-Guerrero & Francisco Barona-Gómez*

Table of Contents

Figure S1. EvoMining heatplot of expansions from EF that belong to scytonemin BGC

Figure S2. Copy number distribution of EF in scytonemin BGC with an origin in central pathways

Figure S3. TrpA EvoMining tree in Cyanobacteria

Figure S4. TrpB EvoMining tree in Cyanobacteria

Figure S5. TrpC EvoMining tree in Cyanobacteria

Figure S6. TrpD EvoMining tree in Cyanobacteria

Figure S7. TrpE/G EvoMining tree in Cyanobacteria

Figure S8. AroB EvoMining tree in Cyanobacteria

Figure S9. ALS EvoMining tree in Cyanobacteria

Figure S10. Genome size by order in Actinobacteria, Cyanobacteria and Archaea

Figure S11. EvoMining expansion and recruitment results over 42 EF from conserved metabolism

Table S1. Copy number in 42 common EF: Actinobacteria, Cyanobacteria, Pseudomonas, Archaea

Figure S12. Glutamate dehydrogenase EvoMining trees in all lineages

Table S2. EF in scytonemin BGC in the Enzyme DB

Table S3. Links of interactive EvoMining trees

Figure S13. Glutamate dehydrogenase EvoMining trees by cofactor NAD/NADP

Table S1. Function and average copy number in enzyme families in Enzyme DB

Key	Enzyme Family	*Average copy per genome in each database*				***Maximum***	**Minimum**
		Actino bacteria	***Cyano bacteria***	***Pseudo monas***	***Archaea***		
A1	Acetylornithine aminotransferase	8.24	2.72	12.03	3.12	Pseudomonas	Archaea
B1	Glutamine synthetase	3.21	1.17	6.46	1.29	Pseudomonas	Cyanobacteria
C1	Anthranilate synthase component 1	2.58	2.01	2.42	1.1	Pseudomonas	Archaea
D1	Fumarate hydratase	1.93	0.92	3.61	0.45	Pseudomonas	Archaea
E1	Acetolactate synthase large subunit	4.81	1.87	4.69	1.97	Actinobacteria	Cyanobacteria
F1	Aspartate transaminase	3.38	2.52	5.53	2.96	Pseudomonas	Archaea
G1	Imidazole glycerol phosphate synthase H	1.90	2.05	2.89	1.81	Pseudomonas	Archaea
A2	Dihydro picolinate synthase	2.09	0.9	3.1	1.01	Pseudomonas	Cyanobacteria
B2	Dihydroxy acid dehydratase	1.68	0.96	2.73	0.75	Pseudomonas	Archaea
C2	Diaminopimelate decarboxylase	1.68	1.02	2.43	0.54	Pseudomonas	Archaea
D2	Cysteine synthase	2.84	2.34	2.71	0.89	Pseudomonas	Archaea
E2	Acetylglutamate kinase	0.98	0.92	1.84	0.41	Pseudomonas	Archaea
F2	3-isopropylmalate dehydrogenase	1.43	1.05	2.45	1.76	Pseudomonas	Cyanobacteria
G2	Citrate synthase	2.14	0.92	1.97	0.71	Actinobacteria	Archaea
A3	Glycine hydroxymethyltransferase	1.76	0.89	2.24	0.91	Pseudomonas	Archaea
B3	Phosphoribosyl isomerase A	0.95	1.53	1.81	1.12	Pseudomonas	Archaea
C3	Fumarate reductase iron sulfur subunit	1.81	0.47	1.04	0.57	Actinobacteria	Cyanobacteria
D3	Glutamate 5 semialdehyde dehydrogenase	0.96	1.43	1.05	0.26	Cyanobacteria	Archaea
E3	Glutamine 2 oxoglutarate aminotransferase	1.18	0.23	1.46	0.5	Pseudomonas	Cyanobacteria
F3	3 dehydroquinate synthase	1.17	1.22	1.13	0.2	Pseudomonas	Archaea
G3	Pyruvate kinase	1.34	1.44	1.6	0.6	Pseudomonas	Archaea
A4	Imidazoleglycerol phosphate dehydratase	0.94	0.87	0.94	0.74	Archaea	Cyanobacteria
B4	Glutamate synthase	0.96	1.08	0.96	0.19	Cyanobacteria	Archaea
C4	Isopropylmalate isomerase large subunit	1.21	1.03	1.2	1.83	Archaea	Cyanobacteria
D4	Ornithine carbamoyltransferase	1.15	0.9	1.63	1.23	Pseudomonas	Cyanobacteria
E4	Argininosuccinate lyase	1.05	0.92	1.27	0.75	Actinobacteria	Archaea
F4	N acetylglutamate synthase	0.99	0.93	0.97	0.3	Pseudomonas	Archaea
G4	glutamate dehydrogenase	0.74	0.56	0.65	1.23	Archaea	Cyanobacteria
A5	Pyrroline 5 carboxylate reductase	0.97	0.89	0.99	0.39	Pseudomonas	Archaea
B5	Threonine synthase	1.67	1.48	1.21	1.79	Archaea	Pseudomonas
C5	Tryptophan synthase alpha	1.00	1.03	1	0.56	Cyanobacteria	Archaea
D5	Indole-3-glycerol phosphate synthase	1.10	1.06	1.02	0.67	Actinobacteria	Archaea
E5	Ribose phosphate pyrophosphokinase	1.23	0.93	1	0.84	Actinobacteria	Archaea
F5	Histidinol dehydrogenase	1.03	1.06	1	0.75	Cyanobacteria	Archaea
G5	Argininosuccinate synthase	1.06	0.92	0.99	0.77	Actinobacteria	Archaea
A6	Enolase	1.18	0.93	1.05	0.89	Actinobacteria	Archaea
B6	N acetyl gamma glutamyl phosphate reductase	1.02	0.93	0.97	0.75	Actinobacteria	Archaea
C6	Phosphoribosylanthranilate isomerase	1.12	0.87	0.98	0.48	Actinobacteria	Cyanobacteria
D6	Tryptophan synthase beta	1.22	1.02	1.08	1.22	Actinobacteria	Cyanobacteria
E6	Ketoacid reductoisomerase	1.00	0.89	0.98	0.8	Pseudomonas	Archaea
F6	Phosphoglycerate kinase	0.98	0.92	0.96	0.87	Pseudomonas	Archaea
G6	Anthranilate phosphoribosyltransferase	0.95	0.88	0.97	0.89	Pseudomonas	Cyanobacteria

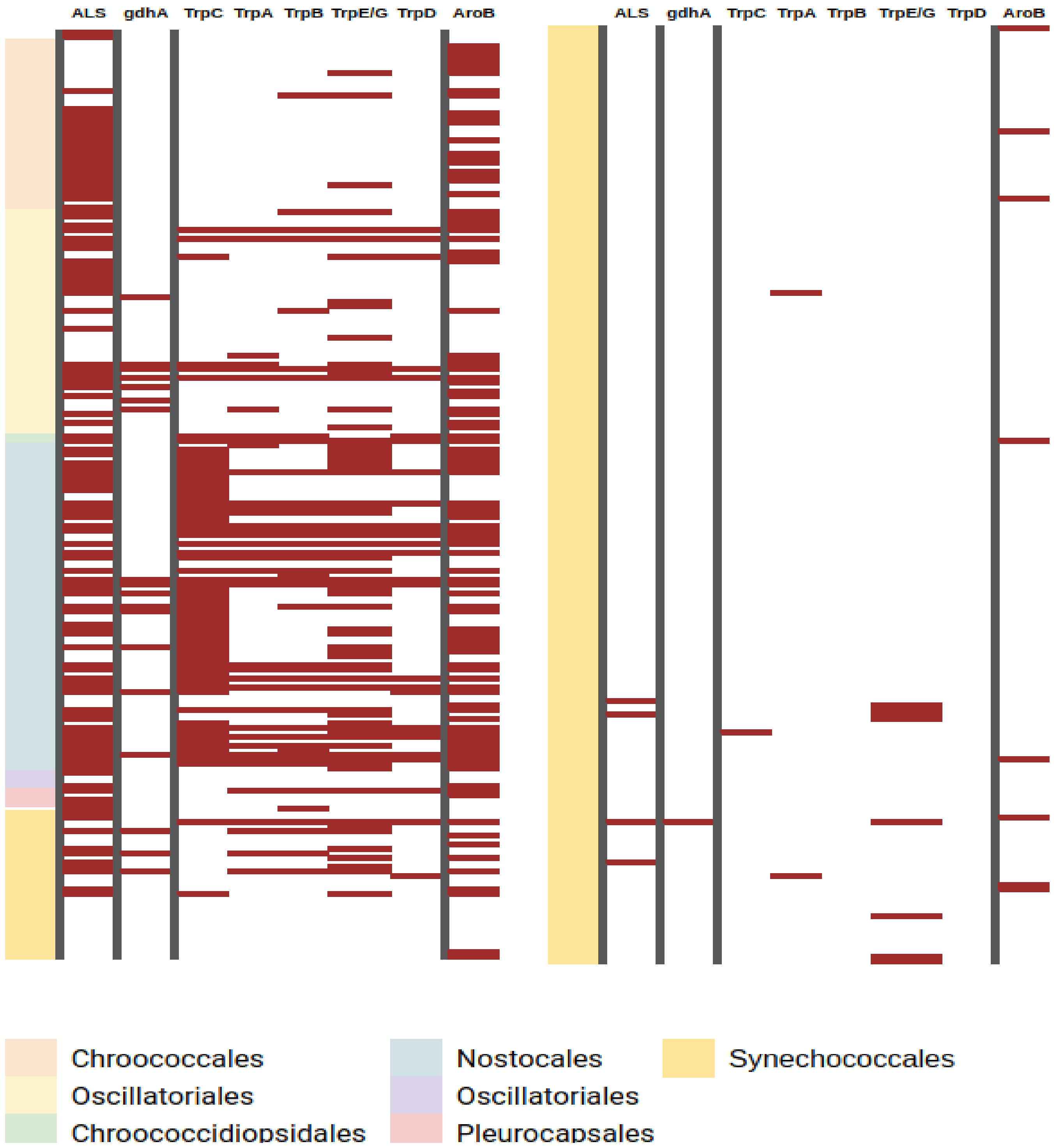

Fig S1. Expansion-and-recruitment heat plot of enzyme families with and origin in conserved metabolism and fate in scytonemin BGC. Rows represent organisms and columns show enzyme families. Coordinates where an organism possess several extra copies beyond the medium plus a standard deviation of an enzyme family are marked in red. The heatplot shows results for the Cyanobacteria phylum with an origin in conserved metabolism and fate in scytonemin BGC. GDH and ALS are the biosynthetic genes in the scytonemin BGC. In this example of scytonemin EF note that there are cases, e.g. in Nostocales, were the synthesis families GDH and ALS (the

corresponding scyB and scyA in specialized metabolism) are expanded, while the precursor families TrpA, TrpB, TrpC or TrpE/G are not expanded.

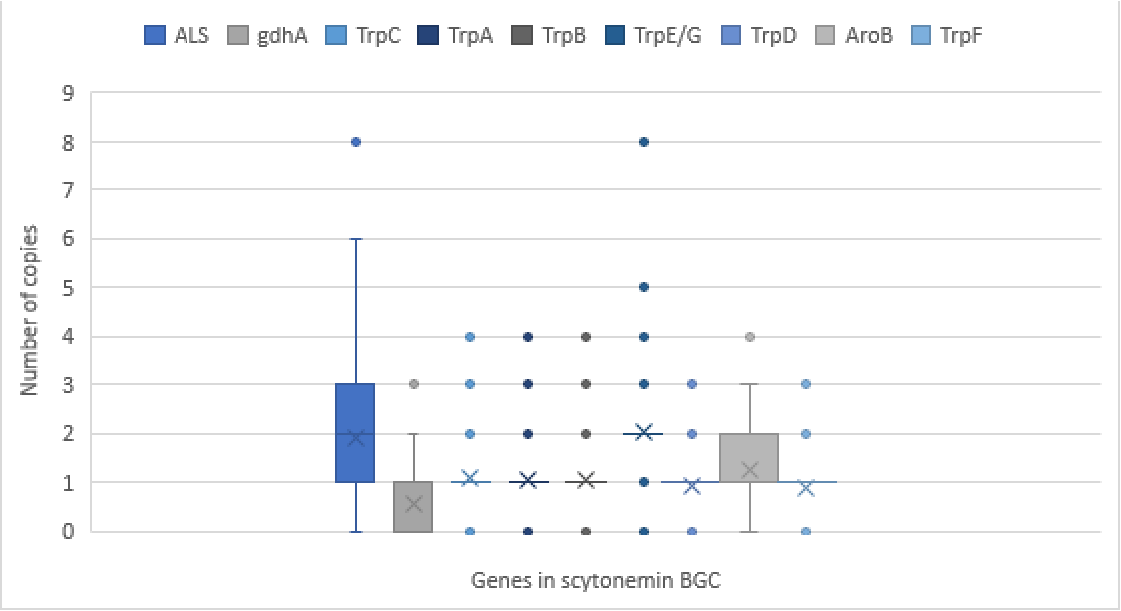

b)

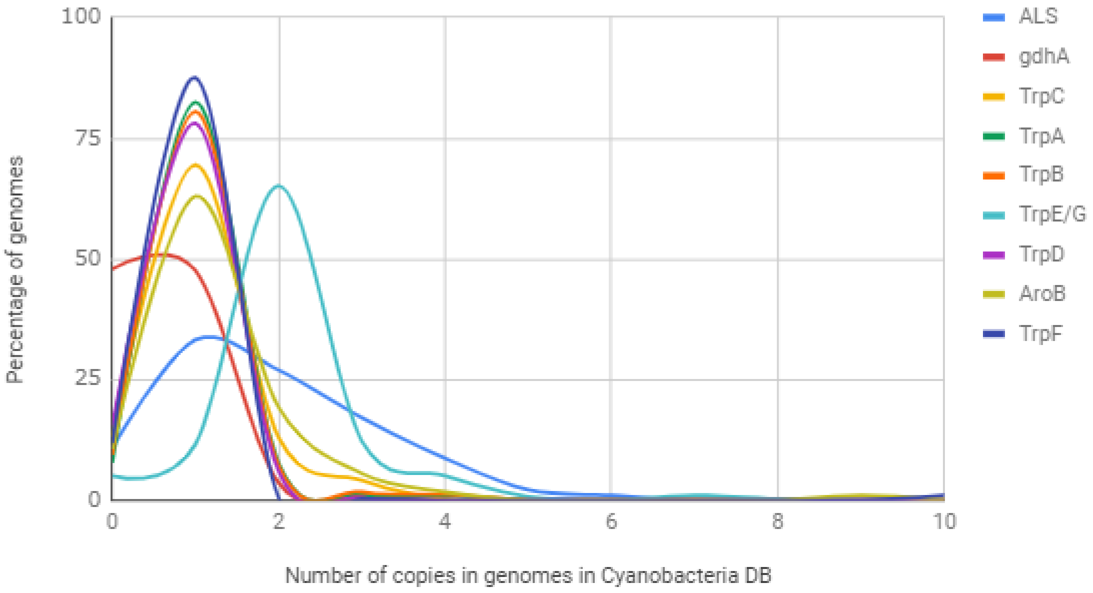

Fig S2. Distribution of copy number of functions in scytonemin BGC and/or Trp operon. **a)** Average copy number is one in TrpF operon except in TrpE/G, where the average is two. However, there is few variations in the copy number of these enzyme families. Note that ALS, GDH and AroB show variance. **b)** EF in scytonemin BGC has between 50% and 80% of genomes with at least one copy of the family. The mode is 1 in all EF except in TrpE/G, where the mode is 2. Expansions are reflected in the second pick at the right of the mode. TrpF does not show expansions in this graph.

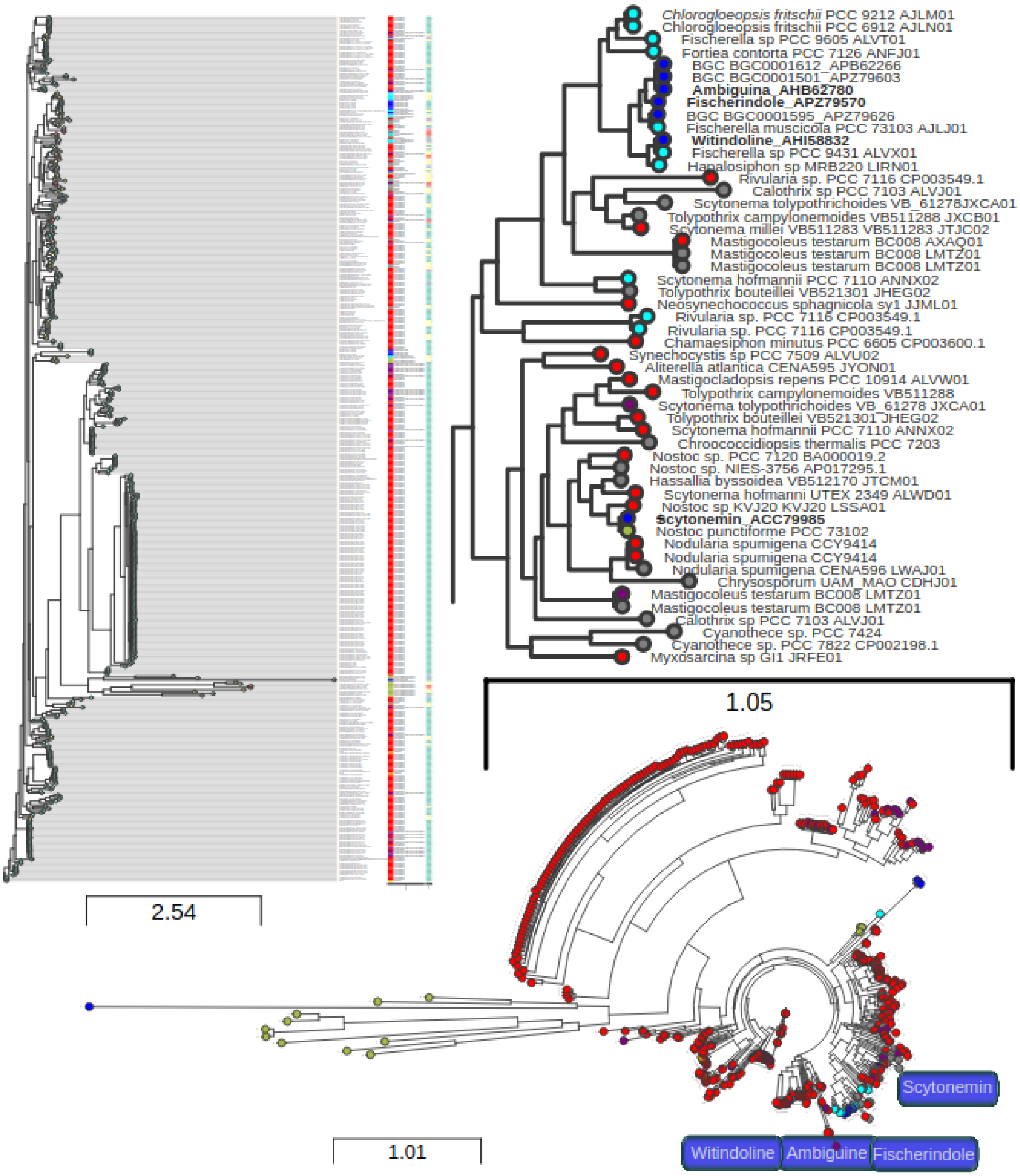

Fig S3. TrpA EvoMining tree. TrpA has an average copy number of 1.03. In the vertical tree of the left copy number is colored in the second column, genomes with one copy are marked in green, two copies in yellow, three copies violet and four in pink. Most of the genomes contain only one copy, but there is a zone in the tree, between ambiguine and scytonemin recruitments were two copies are predominantly observed.In the tree of the bottom we can observed that the low copy number on average is reflected in the fact that EvoMining tree show only a few expansions and recruitments close to scytonemin *trpA* recruitment. Welwitindolinone (labeled as witindoline), Ambiguine and Fischerindoline are other recruitments in TrpA tree.

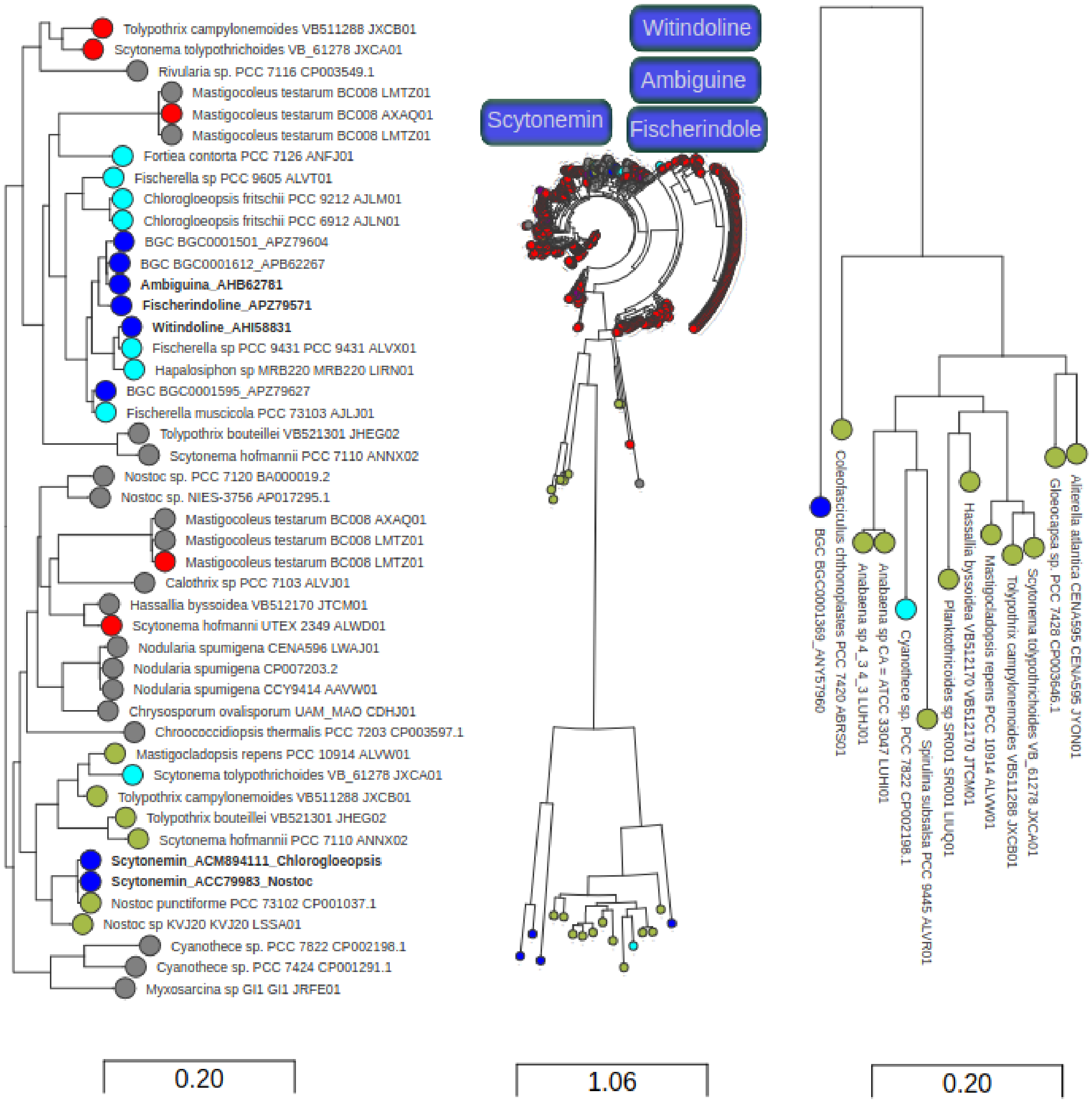

Fig S4. TrpB EvoMining tree. TrpB has an average copy number of 1.02 in Cyanobacteria but is present in only 90% of the genomes, this allos trpB family to present expansions in 10% of the genomes that has at least one copy. In the right are shown expansions and recruitments close to the scytonemin *trpB* recruitment. Welwitindolinone (labeled as witindoline), Ambiguine and Fischerindoline are other recruitments in TrpB tree. There is another divergent branch with many MIBiG recruitments.

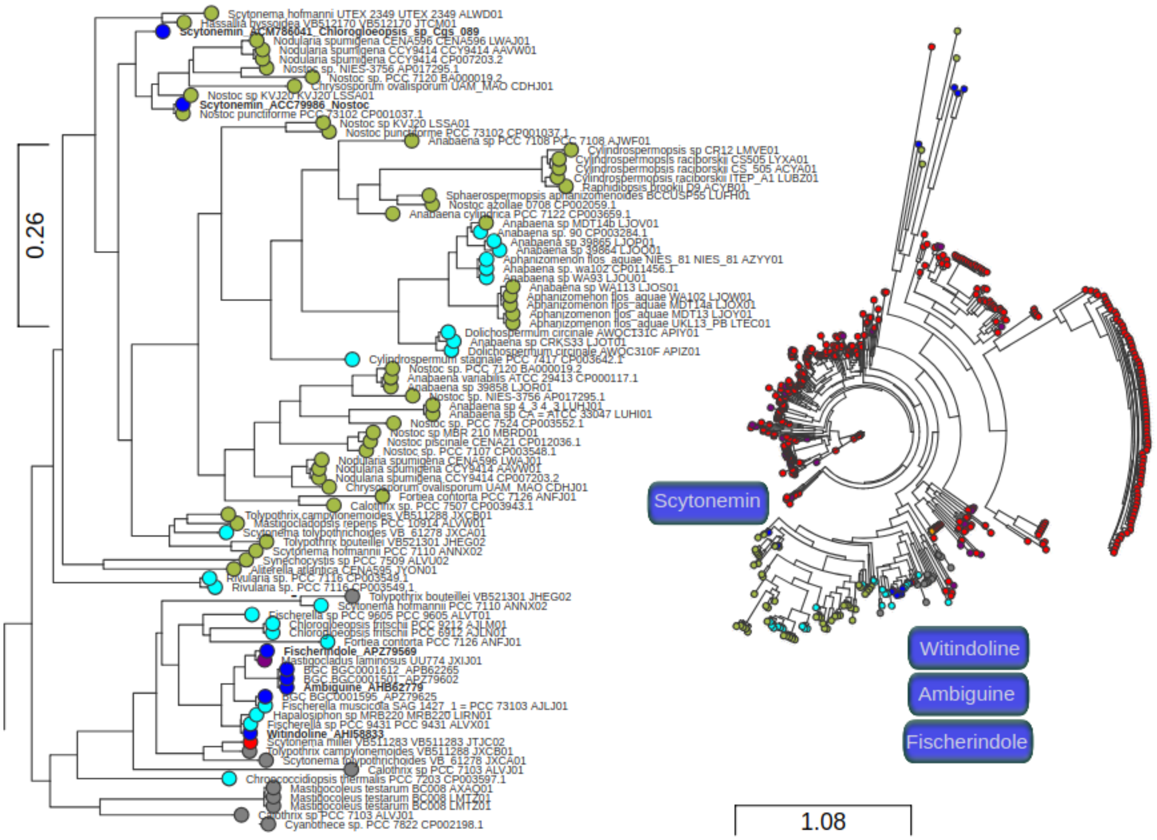

Fig S5. TrpC EvoMining tree. TrpC has an average copy number of 1.06 in Cyanobacteria but is present in only 86% of the genomes. To reach the 1.06 in average in copy number, some of the 86% of genomes that contain TrpC must have more than one copy. TrpC family present expansions in 20% of genomes with at least one copy. In the left is shown a branch populated of EvoMining hits close to the scytonemin *trpC* recruitment. Welwitindolinone (labeled as witindoline), Ambiguine and Fischerindoline are other recruitments in TrpC tree.

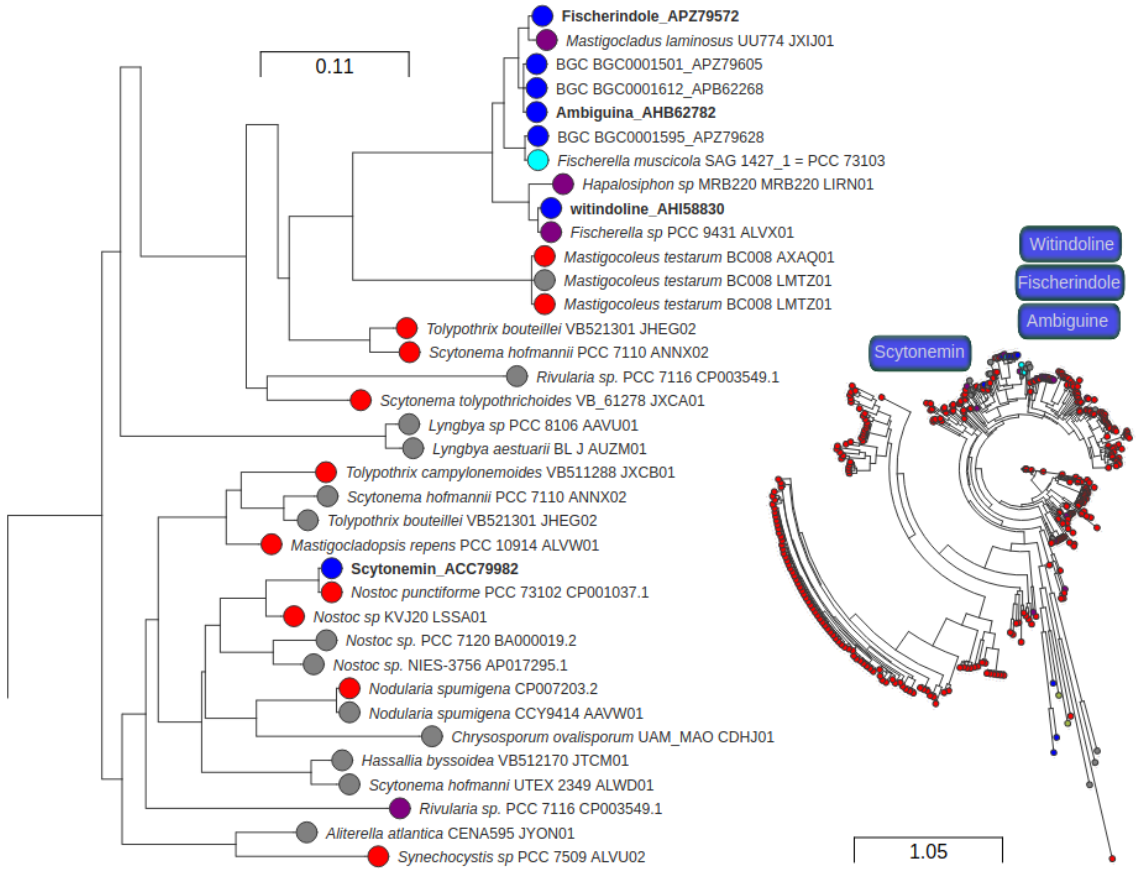

Fig S6. TrpD EvoMining tree. TrpD has an average copy number of 1 in Cyanobacteria and present in only 84.6% of the genomes. To reach the 1 in average in copy number, some of the 84.6% of genomes that contain TrpD must have more than one copy. The mode in this family is also 1 and there are only 7.6% of organisms that surpass this mode. In the left is shown a branch poorly populated of EvoMining hits close to the scytonemin *trpD* recruitment. Welwitindolinone (labeled as witindoline), Ambiguine and Fischerindoline are other recruitments in TrpC tree.

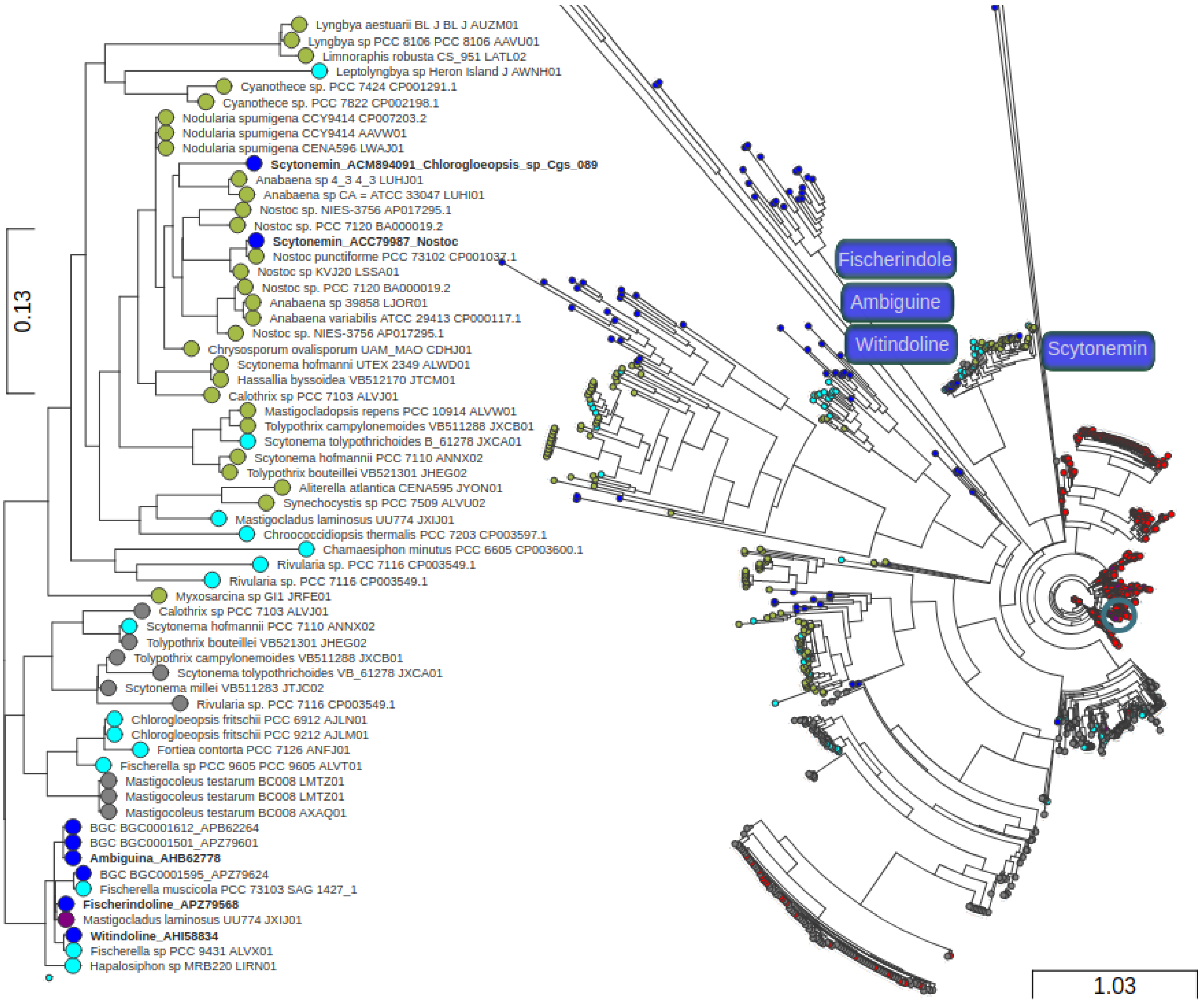

Fig S7. TrpE/G EvoMining tree. TrpE/G is a very expanded family in Cyanobacteria. It is present in 94.9% of the genomes, it has as average copy number of 2.1 and a mode of 2. There is a 19.2 % TrpE/G organisms more than 2 copies. The branch of scytonemin *trpE/G* recruitment is full of extra copies marked as EvoMining hits. Welwitindolinone (labeled as witindoline), Ambiguine and Fischerindoline are also recruitments in TrpE/G tree.

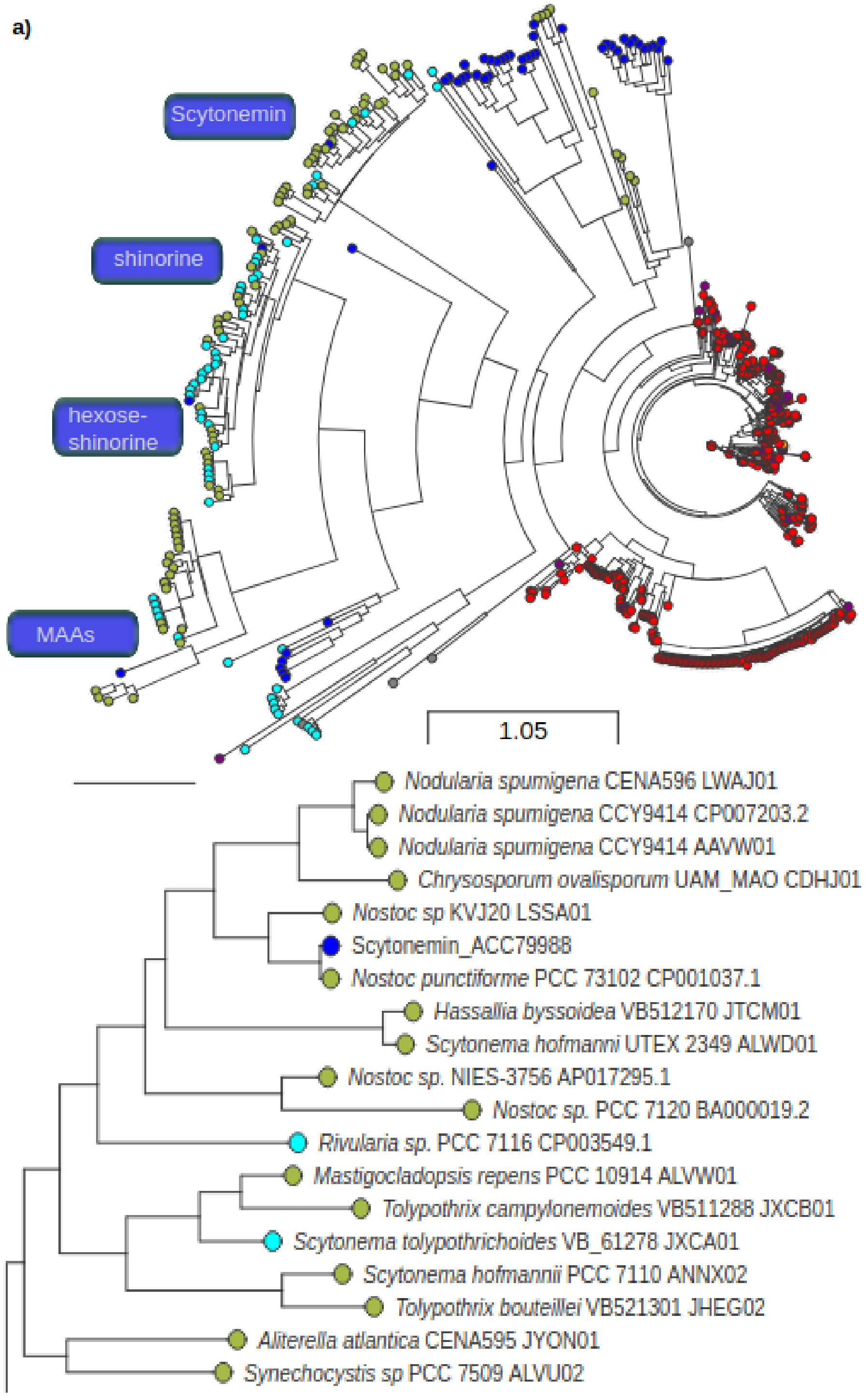

Fig S8. AroB EvoMining tree. The average copy number of this family is 1.3 it has a mode of one with 29.5% of the genomes surpassing this threshold. These characteristics are reflected in three branches full of extra copies marked either as antiSMASH or EvoMining hits. The first branch from bottom to top has MAAs as recruitment, the second branch was recruited for shinorine and the third for scytonemin. These three natural products MAAs, shinorine and scytonemin are sunscreens. AroB does not have Welwitindolinone, Ambiguine or fischerindoline as recruitments as the families from Trp operon.

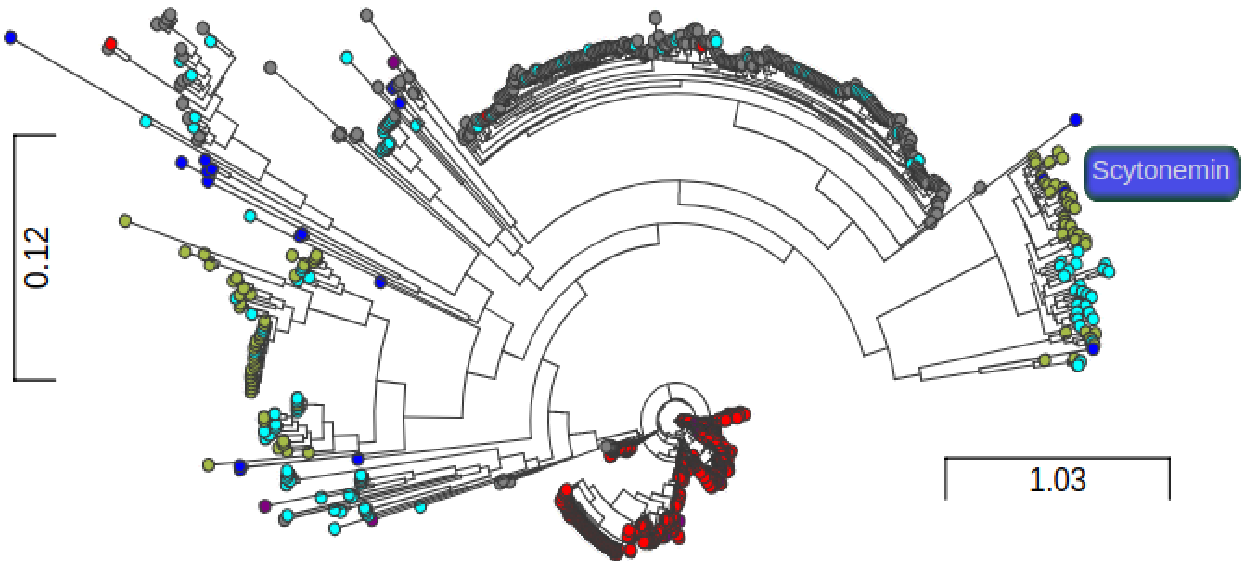

Fig S9. ALS EvoMining tree. This family has as 2.1 as average copy number and a mode of 1 which suggests that many organisms has extra copies. In fact there are 62.8 % genomes above the mode. The branch that contains *scyA* recruitment is full of EvoMining hits.

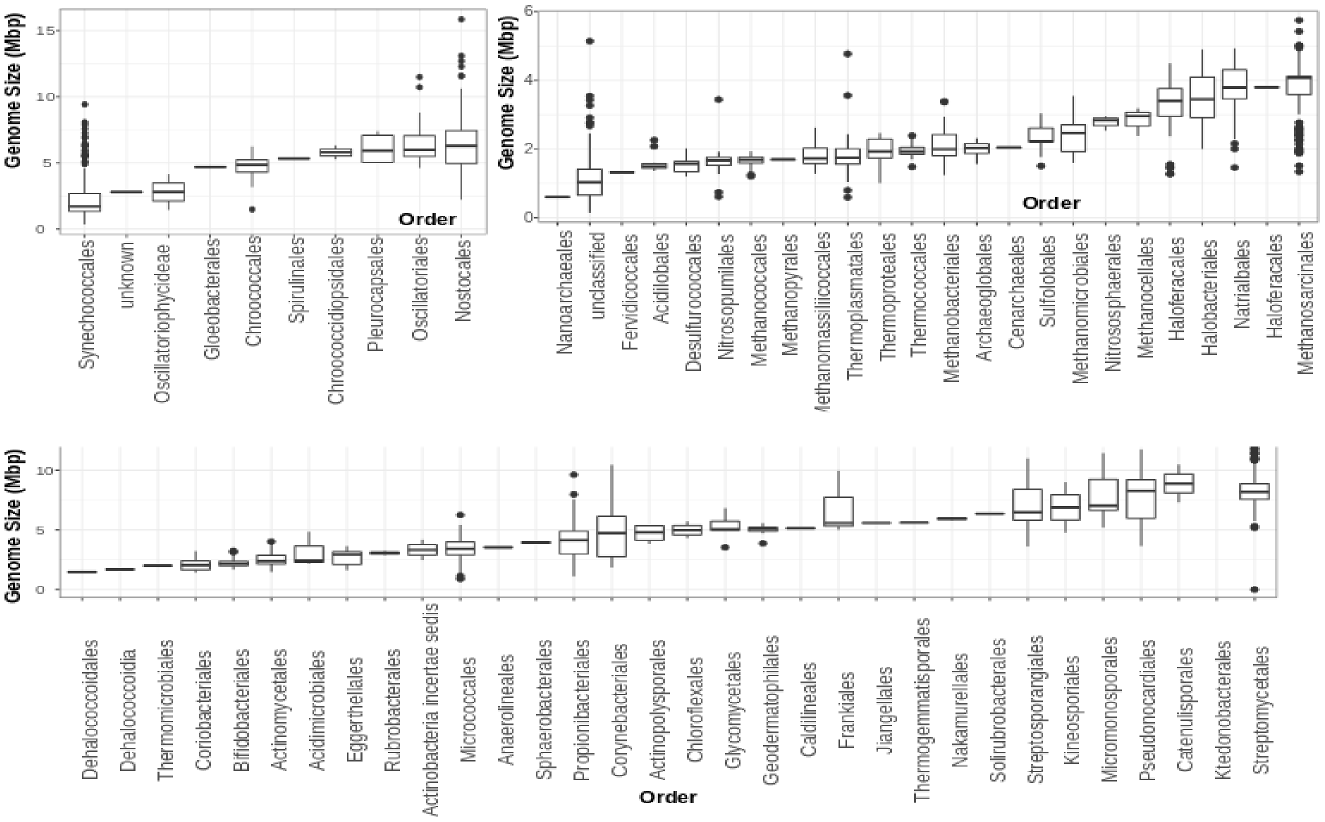

Fig S10. Genome size by order/family in Actinobacteria, Cyanobacteria and Archaea. Actinobacteria and Cyanobacteria has some genomes with sizes greater than 6Mbp which is the maximum shown in Archaea. Average genome size in *Pseudomonas* genus is 5.8 MBbp with a maximum of 7.6Mbp among the genomes used in this work (not shown in the figure).

Table S2. Expansions of EF from scytonemin BGC and TrpF. This table shows percentages only considering organisms with at least one copy.

Key	Function	Gene	Average and percentages calculated in genomes with copy number at least 1 (CN>=1)				
			Percentage of genomes in Cyanobacteria with CN>=1	Average CN	Expansions percentage	Mode	Percentage of organisms above mode
E1	Acetolactate synthase large subunit	*als*	89.1	2.1	11.9	1	62.8
G4	Glutamate dehydrogenase	*gdh*	52.1	1.1	4.5	1	8.7
C5	Tryptophan synthase alpha	*trpA*	92.3	1.1	9.8	1	10.6
D6	Tryptophan synthase beta	*trpB*	90.6	1.1	10.0	1	11.1
D5	Indole-3-glycerol phosphate synthase	*trpC*	86.7	1.2	17.2	1	19.9
G6	Anthranilate phosphoribosyl transferase	*trpD*	84.6	1.0	6.4	1	7.6
C1	Anthranilate synthase component 1	*trpE/G*	94.9	2.1	18.2	2	19.2
C6	Phosphoribosyl anthranilate isomerase	*trpF*	88.2	1.0	0.7	1	0.8
F3	3-dehydroquinate synthase	*aroB*	89.4	1.3	7.1	1	29.5

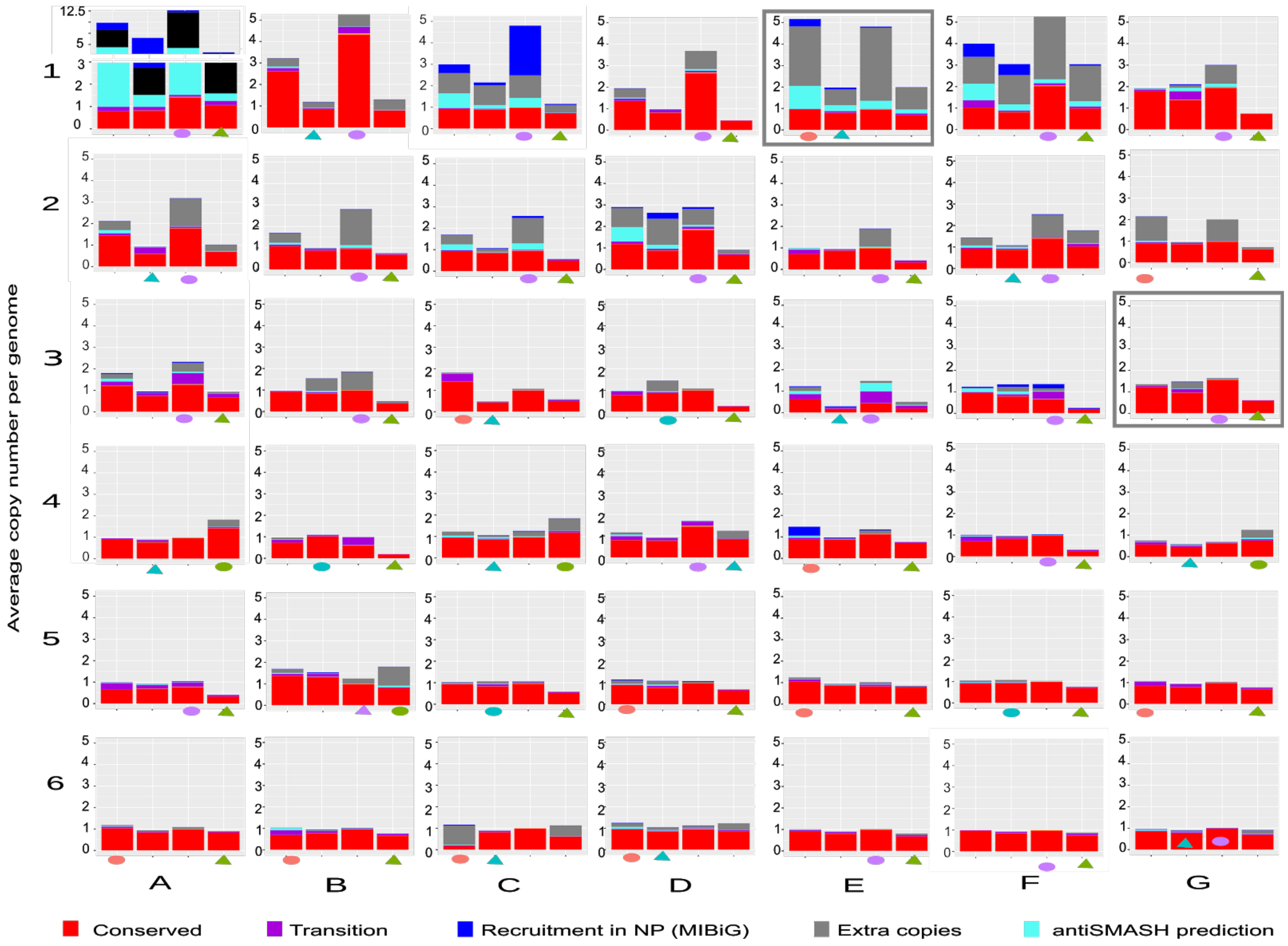

Figure S11. EvoMining profiles of conserved enzymes in selected genomic lineages. Expansions of the 42 conserved EF. Coordinates in the form of letters A-G and numbers 1-6 are shown in this figure to easily localize the family and its properties in Table S1. For every family in the horizontal axes are always shown in order four bars: Actinobacteria, Cyanobacteria, Pseudomonas and Archaea. The color code is as follows: red for conserved metabolism, blue for recruitments annotated at MIBiG, cyan for antiSMASH predictions of specialized metabolism, purple for the intersection between conserved metabolism and antiSMASH predictions, and gray for expansions without known metabolic fate. The letter at the bottom and the numbers at the left are coordinates to facilitate family identification in Table S1. Triangles indicate the lineage with the largest number of copies per genome on average, and circles stands for the least expanded. Although Archaea tends to be the least expanded taxa this tendency reverts in families A4, C4, G4 (GDH) and B5. GDH and ALS in E1, in a box, are the origin of recruitments into scytonemin BGC.

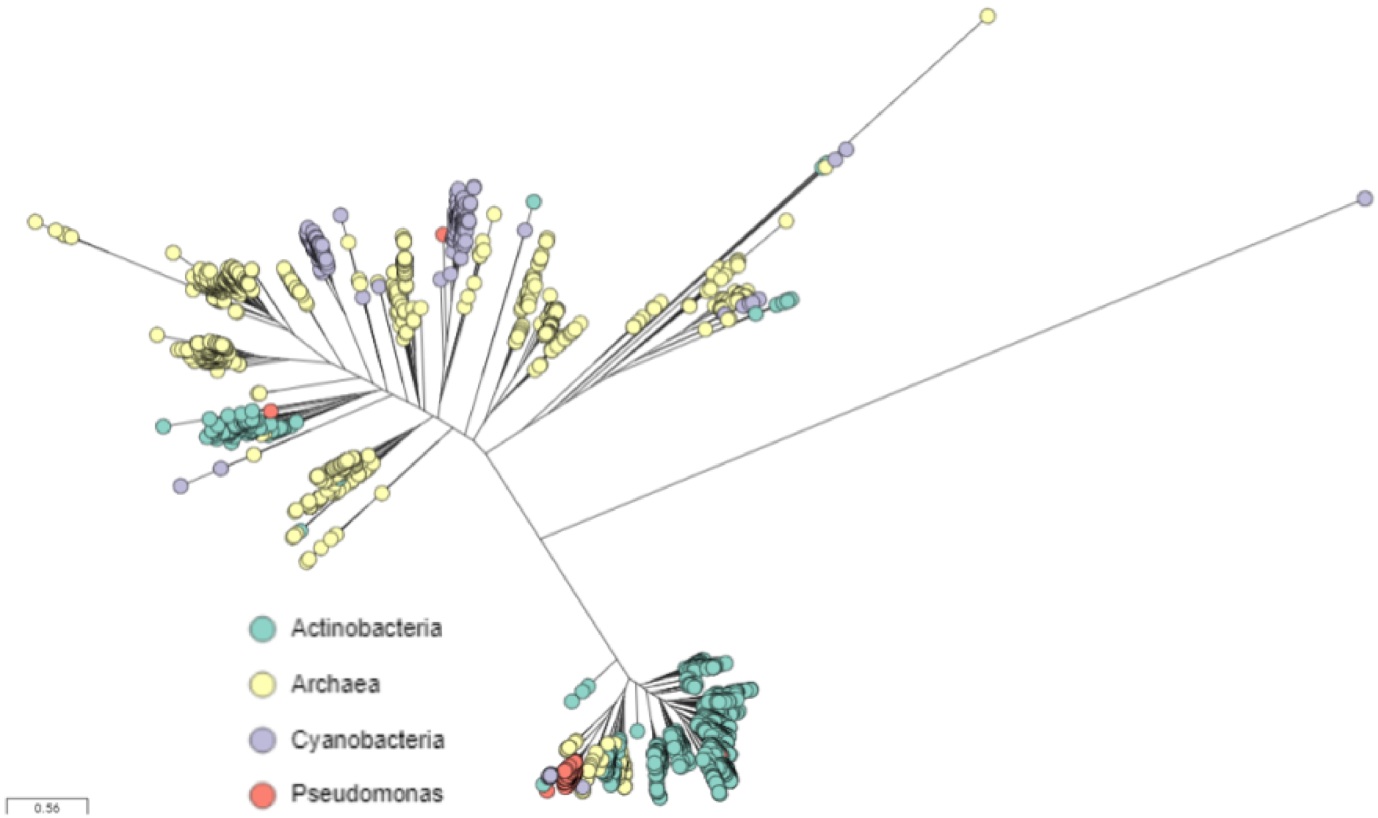

Fig S12 GDH of Actinobacteria, Cyanobacteria, *Pseudomonas* and Archaea (all lineages). Radial reconstruction shows that Actinobacteria (green) is grouped in the bottom but also with a small branch immersed in Archaeal copies (yellow), which denotes the possibility of HGT. Cyanobacteria copies (violet) are in the middle of Archeal copies. *Pseudomonas* is collapsed in one branch surrounded by Archaeal copies close to the major conglomeration of Actinobacteria.

Table S3. Interactive EvoMining trees of scytonemin BGC and TrpF available at Microreact.

Enzyme family	Microreact link [1]
TrpA	https://microreact.org/project/SyZMprKum?tt=cr
TrpB	https://microreact.org/project/H1jW0rFdX?tt=cr
TrpC	https://microreact.org/project/rkN1THFum?tt=cr
TrpE/G	https://microreact.org/project/rkv20SFOm?tt=cr
TrpD	https://microreact.org/project/H1UuQE0qm?tt=cr
AroB	https://microreact.org/project/SkT1Wp_dm?tt=cr
GDH	https://microreact.org/project/HyjYUN7pQ?tt=cr
ALS	https://microreact.org/project/B11HkUtdm?tt=cr
GDH Actinobacteria,Cyanobacteria, *Pseudomonas* and Archaea	https://microreact.org/project/SJw7zVs1V=?tt=rd
TrpF	https://microreact.org/project/BkS5oyjOX?tt=cr
GDH Actinobacteria	https://microreact.org/project/r1lhjVm6X?tt=cr
GDH *Pseudomonas*	https://microreact.org/project/rJPC4EQa7?tt=cr
GDH Archaea	https://microreact.org/project/ByUcvNmaX?tt=cr

[1] Selected features of EvoMining trees (in red) that can be opened in microreact are shown in following figures (Fig S4-S12). Trees not shown are shown black, but these trees can be visualized after coloring them by its metabolic class: conserved, transition, antiSMASH hit, expansion EvoMining hit or MIBiG recruitment, or it can be colored by its copy number. It is also possible to switch labels between the assigned RAST function, the gene ID and the genome name, as EvoMining outputs are compatible with microreact.

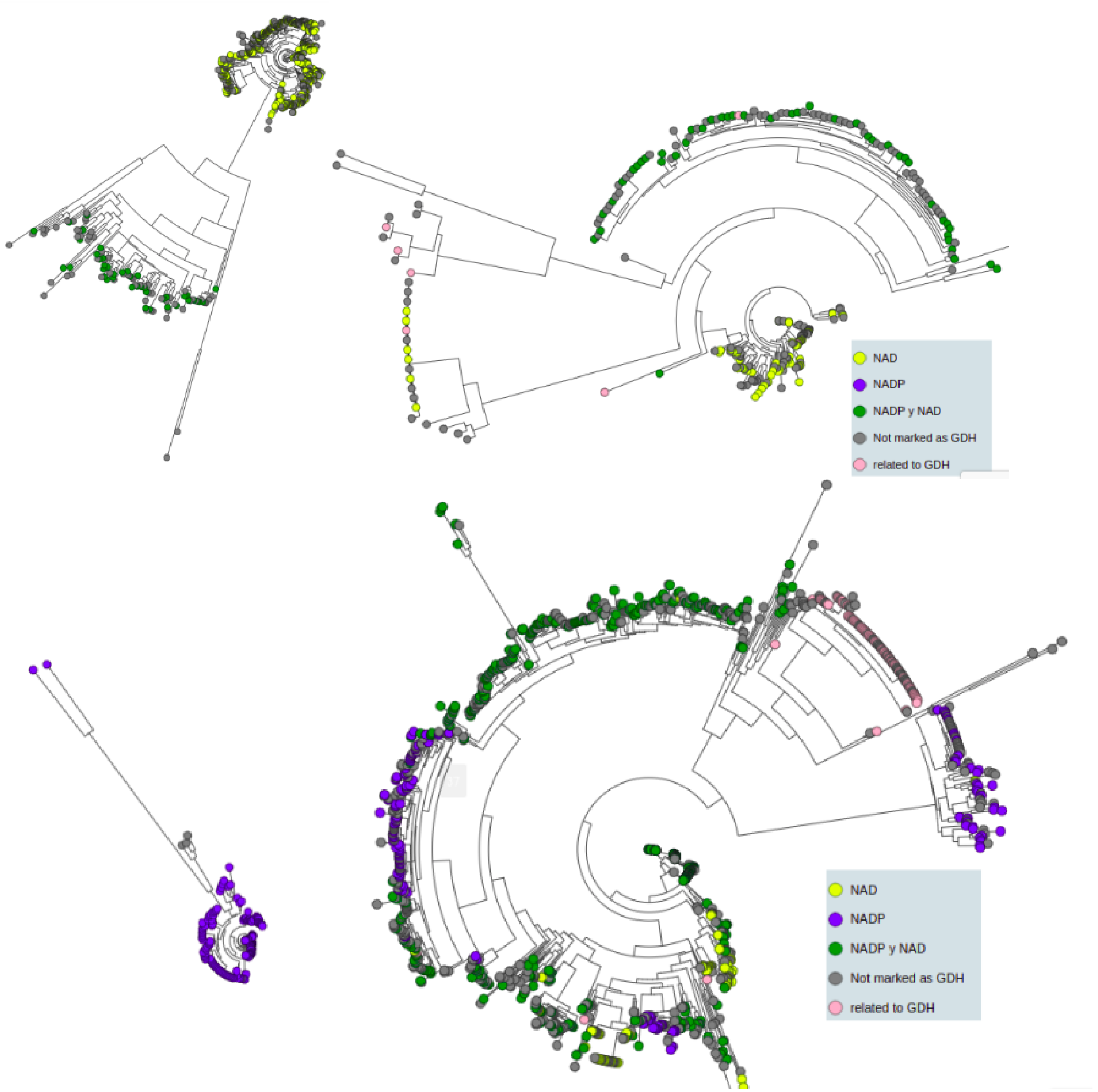

Fig S13. GDH classified by cofactors usage. Pseudomonas shows exclusively NADP dependent enzymes, while Archaea shows a mix between them. Archaea tree is rooted with a dual sequence from the genus *Sulfolobus*. In Cyanobacteria GDH has an average of 1 copy by genomes in genomes that contains at least one copy, the mode is also one in this set and only .8 % of this set is above the mode.
